# ChIP-seq and In Vivo Transcriptome Analyses of the *Aspergillus fumigatus* SREBP SrbA Reveals a New Regulator of the Fungal Hypoxia Response and Virulence

**DOI:** 10.1371/journal.ppat.1004487

**Published:** 2014-11-06

**Authors:** Dawoon Chung, Bridget M. Barker, Charles C. Carey, Brittney Merriman, Ernst R. Werner, Beatrix E. Lechner, Sourabh Dhingra, Chao Cheng, Wenjie Xu, Sara J. Blosser, Kengo Morohashi, Aurélien Mazurie, Thomas K. Mitchell, Hubertus Haas, Aaron P. Mitchell, Robert A. Cramer

**Affiliations:** 1 Department of Microbiology and Immunology, Geisel School of Medicine at Dartmouth, Hanover, New Hampshire, United States of America; 2 Department of Microbiology and Immunology, Montana State University, Bozeman, Montana, United States of America; 3 Bioinformatics Core, Department of Microbiology, Montana State University, Bozeman, Montana, United States of America; 4 Division of Biological Chemistry, Biocenter, Innsbruck Medical University, Innsbruck, Austria; 5 Division of Molecular Biology, Biocenter, Innsbruck Medical University, Innsbruck, Austria; 6 Department of Genetics, Geisel School of Medicine at Dartmouth, Hanover, New Hampshire, United States of America; 7 Department of Biological Sciences, Carnegie Mellon University, Pittsburgh, Pennsylvania, United States of America; 8 Center for Applied Plant Sciences, The Ohio State University, Columbus, Ohio, United States of America; 9 Department of Plant Pathology, The Ohio State University, Columbus, Ohio, United States of America; Washington University School of Medicine, United States of America

## Abstract

The *Aspergillus fumigatus* sterol regulatory element binding protein (SREBP) SrbA belongs to the basic Helix-Loop-Helix (bHLH) family of transcription factors and is crucial for antifungal drug resistance and virulence. The latter phenotype is especially striking, as loss of SrbA results in complete loss of virulence in murine models of invasive pulmonary aspergillosis (IPA). How fungal SREBPs mediate fungal virulence is unknown, though it has been suggested that lack of growth in hypoxic conditions accounts for the attenuated virulence. To further understand the role of SrbA in fungal infection site pathobiology, chromatin immunoprecipitation followed by massively parallel DNA sequencing (ChIP-seq) was used to identify genes under direct SrbA transcriptional regulation in hypoxia. These results confirmed the direct regulation of ergosterol biosynthesis and iron uptake by SrbA in hypoxia and revealed new roles for SrbA in nitrate assimilation and heme biosynthesis. Moreover, functional characterization of an SrbA target gene with sequence similarity to SrbA identified a new transcriptional regulator of the fungal hypoxia response and virulence, SrbB. SrbB co-regulates genes involved in heme biosynthesis and demethylation of C4-sterols with SrbA in hypoxic conditions. However, SrbB also has regulatory functions independent of SrbA including regulation of carbohydrate metabolism. Loss of SrbB markedly attenuates *A. fumigatus* virulence, and loss of both SREBPs further reduces *in vivo* fungal growth. These data suggest that both *A. fumigatus* SREBPs are critical for hypoxia adaptation and virulence and reveal new insights into SREBPs' complex role in infection site adaptation and fungal virulence.

## Author Summary

Despite improvements in diagnostics and antifungal drug treatments, mortality rates from invasive mold infections remain high. Defining the fungal adaptation and growth mechanisms at the infection site microenvironment is one research focus that is expected to improve treatment of established invasive fungal infections. The *Aspergillus fumigatus* transcription factor SrbA is a major regulator of the fungal response to hypoxia found at sites of invasive fungal growth *in vivo*. In this study, new insights into how SrbA mediates hypoxia adaptation and virulence were revealed through identification of direct transcriptional targets of SrbA under hypoxic conditions. A major novel finding from these studies is the identification of a critical role in fungal hypoxia adaptation and virulence of an SrbA target gene, *srbB*, which is also in the SREBP family. SrbB plays a major role in regulation of heme biosynthesis and carbohydrate metabolism early in the response to hypoxia. The discovery of SrbA-dependent regulation of *srbB* gene expression, and the target genes they regulate opens new avenues to understand how SREBPs and their target genes mediate adaptation to the *in vivo* infection site microenvironment and responses to current antifungal therapies.

## Introduction

Invasive fungal infections have increased in frequency due to a substantial rise in the number of immune compromised patients [1]–[3]. In particular, the filamentous fungal pathogen *Aspergillus fumigatus* is a major cause of morbidity and mortality in patients undergoing immunosuppressive therapy for organ transplants and/or cancer treatment [2], [4], [5]. Treatment options for invasive aspergillosis (IA) remain limited and associated mortality rates are high [6]. Epidemiological studies suggest that over 200,000 cases of aspergillosis occur worldwide on an annual basis, and there is general consensus that disease caused by *A. fumigatus* is under diagnosed [2]. It is clear that new insights into the pathophysiology of this too often lethal disease are needed to develop new diagnostic and treatment strategies to improve patient outcomes. Along these lines, investigating fungal growth *in vivo* at infection site microenvironments is an important area of research with significant therapeutic potential.

Observations from human IA cases and recent discoveries in murine models of invasive pulmonary aspergillosis (IPA) indicate that the infection microenvironment is characterized in part by low oxygen availability (hypoxia) [7]–[10]. Hypoxia is associated with poor clinical outcomes for many human diseases, but its impact on invasive fungal infections remains understudied [11]–[16]. One hypothesis is that hypoxia promotes fungal virulence through induction of a fungal metabolic, or bioenergetics, program that contributes to host damage. In support of this hypothesis, fungal genes required for hypoxic growth are generally critical for fungal virulence in murine models, and airway ischemia was recently shown to promote *A. fumigatus* invasion in an orthotropic tracheal transplant model of *Aspergillus* infection [15]–[20].

One fungal gene family required for hypoxia adaptation and growth is the sterol regulatory element binding protein family (SREBP) [21]. First identified in mammals, two distinct mammalian SREBP genes exist, SREBP-1 and SREBP-2. SREBP-1 produces two isoforms, SREBP-1a and SREBP 1-c, derived from alternative splicing of the first exon [22]. These SREBP-1 isoforms share the same DNA-binding domain, the sterol regulatory element (SRE), and have been shown to mainly regulate lipid metabolism, whereas SREBP-2 predominantly regulates cholesterol metabolism [23]. Recent genomic approaches have yielded new insights into mammalian SREBP functions through identification of novel target genes. In mice, ChIP-seq analyses revealed that SREBP-1c binds upstream of genes associated with lipid biosynthesis, insulin dependent pathways, carbohydrate metabolism, and additional novel gene ontology (GO) categories including intracellular protein trafficking, cell proliferation and differentiation, and apoptosis [24]. ChIP-seq analysis of SREBP-2 target genes in murine hepatic chromatin revealed new roles for this protein in apoptosis and autophagy [25]. Thus, it has been suggested that SREBPs are necessary for coordination of the cellular nutritional state and transcriptional activation by interacting with different cofactors in response to dynamic nutritional microenvironments [26].

With regard to SREBP function in fungi, a seminal study in the fission yeast *Schizosaccharomyces pombe* identified a molecular link between SREBP function and fungal hypoxia adaptation and growth [27]. In this organism, the SREBP bHLH domain containing protein, Sre1, has been identified as a primary regulator of anaerobic gene expression [28]. Accordingly, an *sre1* deletion mutant is unable to grow in anaerobic conditions confirming an indirect molecular mechanism of oxygen sensing through Sre1 mediated monitoring of ergosterol levels [27], [29]. Sre1 proteolytic cleavage requires an E3 ligase, which is distinct from the mammalian SREBP cleavage mechanism utilizing site-1 and site-2 proteases [27]–[35]. Subsequent studies in the human pathogenic yeast *Cryptococcus neoformans* and filamentous fungus *A. fumigatus* identified a critical role for fungal SREBPs and associated regulatory factors in hypoxia adaptation, iron homeostasis, azole drug responses, and importantly fungal virulence [18], [19], [36]–[39].

The molecular basis for fungal SREBPs' role in virulence remains to be fully elucidated. Identification of putative SREBP target genes through genome-wide gene expression analyses indicates that fungal SREBPs are important regulators of ergosterol metabolism and iron uptake [18], [21], [38], [39]. In *A. fumigatus*, microarray analyses of gene expression in the presence and absence of SrbA in hypoxic environments revealed significant changes in transcript levels of approximately 12% of the genes in the genome [36]–[38]. Many of the gene transcript levels that are affected by SrbA are associated with biological processes induced by hypoxia in wild type *A. fumigatus* including: ergosterol biosynthesis, iron homeostasis, cell wall biosynthesis, amino acid biosynthesis, general carbon metabolism, and the GABA shunt [40]. However it remains unclear which hypoxia induced genes are directly SrbA dependent. Understanding fungal SREBP function is further complicated by the presence of at least two SREBPs in many of the fungal genomes queried to date [21], [27], [36], [41], [42]. Data also suggest that additional SREBP interacting partners are required for proper modulation of sterol levels in several organisms [21], [26], [43].

Given the little we know of direct fungal SREBP target genes, and the differing transcriptional networks that organisms utilize to respond to similar microenvironments, we sought to precisely define the SrbA-mediated transcriptome (regulon) of *A. fumigatus* in response to hypoxia. In order to definitively delineate the SrbA hypoxia regulon, we utilized a multi-faceted approach to validate not only direct transcriptional targets of SrbA, but also the putative significance of these targets during an invasive pulmonary infection. In addition to identifying a role for *A. fumigatus* SrbA in direct transcriptional regulation of novel genes involved in the fungal hypoxia response, we identified and functionally characterized a second SREBP family member, designated SrbB, which is a direct transcriptional target of SrbA in hypoxia. Together, SrbA and SrbB regulate and co-regulate genes critical for fungal metabolism, virulence, and responses to antifungal drugs. These results place SrbA and SrbB as central transcriptional regulators of fungal metabolic responses required for *in vivo* fungal growth and host damage. Consequently, further characterization of the pathways and networks regulated by SrbA-SrbB is expected to promote a novel research direction aimed at inhibiting the function of this *in vivo* associated fungal genetic network to improve IPA prognosis.

## Results

### ChIP-seq Analysis Identifies a Core Set of Novel SrbA Target Genes in Response to Hypoxia

**Figure 1:**
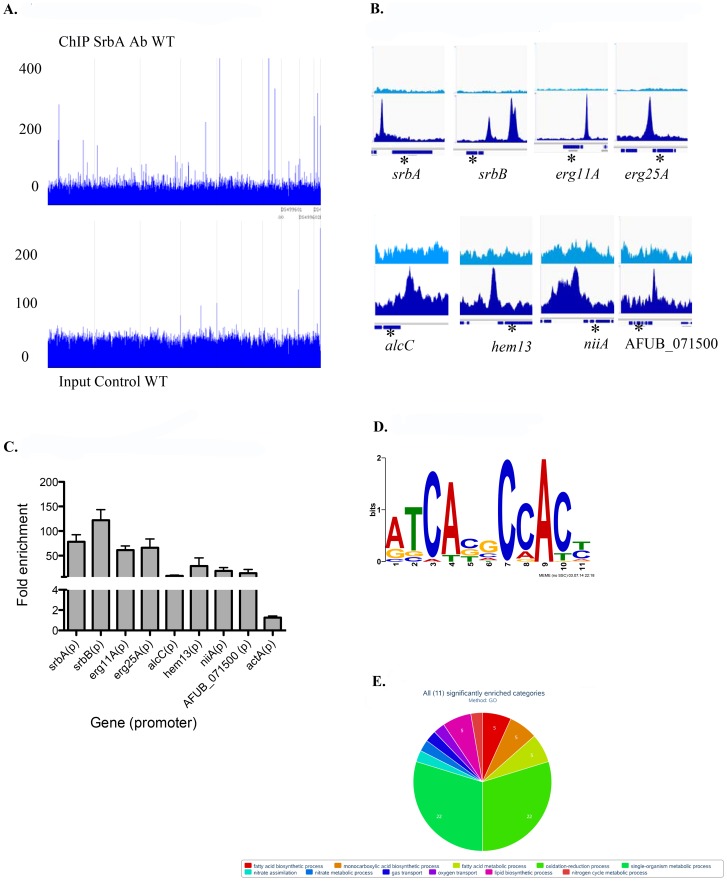
Genome wide ChIP-seq Analysis Identifies Direct Transcriptional Targets of SrbA in Hypoxic Conditions. (A). Genome scale view of ChIP-seq data for one 4-hour ChIP sample. Blue lines rising above background are peaks identified as an excess of sequence fragments aligning to the genome of *A. fumigatus* sequenced strain A1163. The genome-wide view of the ChIP-seq reads aligned to the A1163 genome reveals strong visible peaks in the SrbA antibody ChIP sample, with few strong peaks observed in the A1163 wild type input control sample. Grey lines demarcate genome scaffolds. (B). ChIP-seq peaks for selected genes: *srbA, srbB, erg 11A* and *erg 25A* were among the highest ChIP-seq peaks and have relatively high enrichment (lower panels) vs. input control (upper panels). *alcC, hem13, niiA* and AFUB_071500 show significant, though lower enrichment vs. input control. In each case the peaks are upstream of the translational start site of the indicated genes. Asterisks mark the relevant gene model within each 3 kb region. (C). ChIP qPCR was conducted to validate ChIP-seq results for select targets. Data are presented as the mean and standard error of two biological replicates. The actin promoter (*actA*(p)) was included as a non-specific target. SrbA was enriched on the promoters of all tested genes. (D). SrbA binding motif as identified using MEME. (E). GO pie chart.

The workflow for this study was informed by previous research that assessed the significance of SrbA in hypoxia growth and fungal pathogenesis [36] and subsequent studies looking at transcriptional changes associated with the *A. fumigatus* response to hypoxia [38], [40], [44]. To gain new mechanistic insights into how SrbA mediates these important phenotypes, ChIP-seq analysis using an SrbA specific antibody after 4 hours (two biological samples) and 12 hours (one biological sample) exposure to hypoxia was conducted [37], [38]. The overall number of Illumina 76 base-pair paired end reads used for peak calling among the three sets of ChIP-seq samples were 2992021 and 3345809 for ChIP and input control respectively (Table S1). Reads were aligned to the *A. fumigatus* A1163 genome (Figure 1A) and used for peak calling with the Macs2 program either in aggregate, or one sample at a time [45]. By including input controls (samples with no SrbA antibody) in the analysis with an FDR of 0.05, a core set of 111 peaks corresponding to 97 genes were identified (Table S2). A subset of 30 genes of biological interest associated with SrbA binding events are listed in Table 1. 25 of 30 (83%) of these peaks were located within 1 kb upstream of translational start sites (Table 1). Figure 1B shows examples of peak regions from eight target genes. Using additional independent biological replicates, ChIP-qPCR confirmed SrbA binding to the promoter regions of the selected genes in response to hypoxia (Figure 1C).

**Table 1:** Selected SrbA ChIP Peaks with flanking gene information.

AFUB Gene ID	Flanking gene description	Distance from peak	Peak location: chrom.	Peak location: start	Peak location: end	ChIP-seq fold enrichment	srbA120/WT-120	srbB120/WT-120
AFUB_063960*	14-alpha sterol demethylase Cyp51A/Erg11A	**302**	DS499598	332610	333065	17.9	**−7.25**	−0.119
AFUB_098170*	C-4 methyl sterol oxidase Erg25B	**224**	DS499603	11566	11908	15.3	**−3.49**	−0.18
AFUB_099590*	HLH DNA binding domain protein SrbB	1557, 1742, **307**	DS499603	388883	389504	13.7, 12.5, 10.5	−1.77	**−12.76**
AFUB_084150*	C-4 methyl sterol oxidase Erg25A	**118**	DS499600	1281613	1282279	11.4	**−4.62**	−0.63
AFUB_058270*	pyridoxamine phosphate oxidase	2776	DS499597	2624700	2625119	10.86	**−2.35**	−0.86
AFUB_004350*	cytochrome P450 sterol C-22 desaturase Erg5	1270	DS499594	1211332	1211722	10.1	0.55	**2.79**
AFUB_018340*	HLH transcription factor SrbA	**748**	DS499595	288316	288619	8.37	**−2.64**	0.88
AFUB_047590*	cholestenol delta-isomerase Hyd1	**201**	DS499596	3935791	3936110	8.36	−1.29	**2.75**
AFUB_093140*	sterol delta 5,6-desaturase Erg3B	**316**	DS499602	76613	76863	7.23	**−4.05**	1.12
AFUB_049300*	integral membrane protein	**767**	DS499597	159489	159783	5.77	**−2.00**	0.085
**AFUB_024300**	MFS allantoate transporter	**866**, 1542	DS499595	2137391	2139246	5.67, 4.15	**−2.79**	−1.40
AFUB_091650*	siderochrome-iron transporter Sit1	**605**	DS499601	1492563	1492827	5.58	**−3.56**	**−2.23**
AFUB_067210*	FAD binding domain protein	**291**	DS499598	1171355	1171629	5.25	**−2.76**	−0.0068
AFUB_045810*	protein kinase	2086	DS499596	3485856	3486109	5.03	−1.71	1.97
AFUB_089270*	14-alpha sterol demethylase Cyp51B/Erg11B	**409**	DS499601	799874	800156	4.55	−0.74	1.26
**AFUB_028270**	NADPH-adrenodoxin reductase	2571	DS499595	3221307	3221539	4.44	−0.95	0.69
**AFUB_091500**	stearic acid desaturase SdeA	**419**	DS499601	1452551	1452773	4.26	−0.45	−0.043
AFUB_054020*	serine/threonine protein kinase	1676	DS499597	1482327	1482532	4.24	−1.15	−0.51
AFUB_071490*	RING finger protein	**736**	DS499598	2397368	2397619	4.15	−1.69	−1.34
AFUB_007800*	coproporphyrinogen III oxidase Hem13	**158**	DS499594	2185402	2185624	4.09	**−2.38**	**−3.37**
**AFUB_051900**	flavin dependent monooxygenase	**170**	DS499597	844487	844713	4.08	**−2.598**	0.028
**AFUB_094530**	oxidoreductase (Msc7)	**94**	DS499602	473054	473225	3.66	−1.30	0.26
**AFUB_052690**	molecular chaperone Mod-E/Hsp90	**452**	DS499597	1036233	1036388	3.63	−0.49	0.50
**AFUB_072390**	heat shock protein Hsp30-like	**265**	DS499599	53106	53275	3.44	−1.18	−0.086
AFUB_099650*	flavohemoprotein	**501**	DS499603	401118	401336	3.43	−0.17	−1.27
**AFUB_042870**	cytochrome c oxidase subunit Via	**78**	DS499596	2518125	2518319	3.4	−1.85	−0.902
**AFUB_071500**	iron regulated transporter	**130**	DS499598	2404243	2404154	2.89	−1.74	−0.33
**AFUB_012310**	Nitrate reductase NiiA	**73**	DS499594	3461069	3460941	2.3	−1.00	−1.49
**AFUB_012300**	Nitrate reductase NiaD	**319, 498**	DS499594	3461069	3460521	1.86, 2.3	−0.80	.036
AFUB_053780*	Alcohol dehydrogenase AlcC	**378**	DS499597	1400950	1401098	2.11	**−2.32**	**−12.6**

ID = gene ID for the *Aspergillus fumigatus* A1163 genome, and asterisk (*) indicates this gene was also surveys with Nanostring. Annotation description is based on annotations available. Peak location is the genome location for A1163 genome, including chromosome, start and end of peak. Distance to peak indicates the nucleotide distance from the center of the peak to the 5′ start codon of the indicated gene, bold is within 1 kb. Fold enrichment indicates the fold enrichment across the entire peak; higher values indicate a greater number of aligned ChIP-seq reads. Final 2 columns are RNAseq fold expression changes (Log2) for deletion strains compared to wild type at 2 hours post hypoxia exposure.

An SrbA-bound DNA motif (Figure 1D) was discovered using multiple EM for motif elicitation (MEME) of the identified peaks [46]. The SrbA DNA binding motif was identified as an 11-bp binding region predominated by ATCA in positions 1–4, a cytosine in position 7, and an adenine in position 9. Other positions were more variable, with a predominance of cytosine residues. This DNA motif (5′ (A/G)TCA(T/C/G)(C/G)CCAC(T/C)-3′) is similar to a previously identified SrbA DNA binding motif that was discovered using bioinformatic tools, 5′-ATC(G/A)(T/G)(A/G)(C/T)(G/C)AT-3′ [47]. The sequence TCACNCCAC has been identified in humans as the SRE binding motif [48], [49]. Additionally in *S. pombe*, a motif was defined using MEME to be (A/G)(C/T)C(A/G/T)NN(C/T)(C/T/G)A(C/T), which contains similar conserved residues as our sequence [28]. Recently, the bHLH transcription factor Hms1 in *C. albicans* was found to bind the consensus sequence ATCACCCCAC, which is strikingly similar to the identified motif for SrbA [50]. Using this motif, we analyzed a previously published Δ*srbA* microarray dataset, which revealed that this SrbA DNA binding motif is overrepresented among the differentially expressed genes when comparing wild type to Δ*srbA* further validating the SRE motif [38]. Five of the identified peaks had two-to-three occurrences of the motif, including SrbA itself. The binding of SrbA to its own promoter reveals an autoregulatory positive feedback loop for modulation of *srbA* mRNA levels.

To further determine the biological processes and molecular function of SrbA direct target genes, SrbA target genes were analyzed for gene ontology (GO) and FunCat enrichment with FungiFun2 (Figure 1E, Table S3) [51]. Of the 97 unique SrbA target genes, only 19 were significantly enriched in FunCat categories (P≤0.05) and 33 in GO categories (P≤0.05). Overall, approximately 35% of the SrbA target genes are not currently annotated. Consistent with our previous microarray-based transcriptome analysis, and known functions of yeast and mammalian SREBPs, SrbA is a direct regulator of genes involved in lipid, fatty acid and isoprenoid biosynthesis, which includes ergosterol biosynthesis (Table S3). In addition, *A. fumigatus* SrbA target genes were associated with heme and oxygen binding and nitrate metabolic processes. Taken together, annotated SrbA direct target genes are associated with biological processes impacted by both oxygen and iron limitation that are critical for fungal virulence. These results thus indicate that SrbA is a major transcriptional regulator of fungal metabolism (bioenergetics) in response to hypoxia.

### RNA-seq and Transcript Analysis of Δ*srbA* in Hypoxia Confirms SrbA Target Genes and Associated Biological Functions

To further define SrbA target genes and confirm SrbA regulation of genes with associated SrbA DNA binding events in their promoter regions, RNA-seq analysis of the hypoxia transcriptome of Δ*srbA* was conducted pooling total RNA samples from independent biological cultures in triplicate. A 30 and 120 minute response to hypoxia was examined by comparing Δ*srbA* samples to the same time point as the wild type. Consistent with the previously published microarray and RNA-seq analyses of the *A. fumigatus* hypoxia response, RNA-seq analyses here revealed substantial changes to the transcriptome of wild type and Δ*srbA* in response to hypoxia [38], [40], [44].

Somewhat surprisingly, loss of SrbA had minimal effect on the transcriptome of *A. fumigatus* after 30 minutes exposure to hypoxia, as only 48 genes had transcript levels decrease 4-fold or greater in Δ*srbA* compared to wild type (Table S4) during this time period. Perhaps of interest, these 48 genes were enriched for functions involving post-translational modification of amino acids. Notably, genes involved in ergosterol biosynthesis are generally unaffected at this time point in the absence of SrbA. However, at 30 minutes post-hypoxia exposure, loss of SrbA resulted in transcript level increases for 383 genes compared to wild type. Interestingly, these genes were enriched for degradation of the amino acids tyrosine and phenylalanine. Previously, an analysis of the free amino acid pool in Δ*srbA* in response to iron replete and limiting conditions revealed substantial changes in the amino acid pools of Δ*srbA* [38].

**Figure 2:**
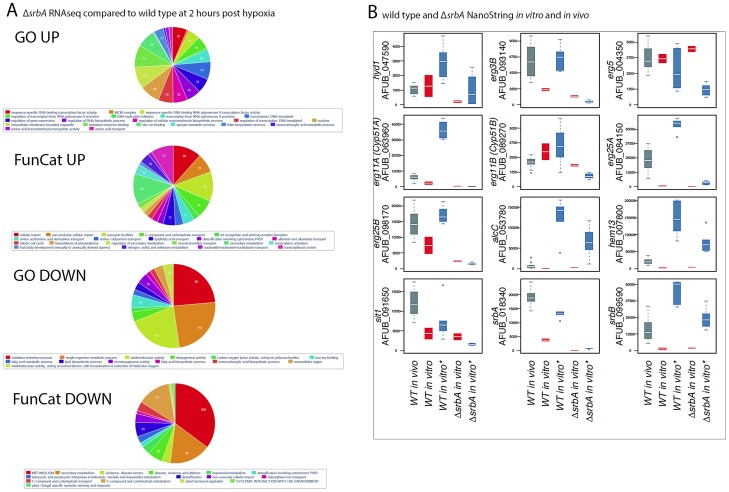
RNA-seq and nCounter Analyses of Δ*srbA* Confirms SrbA Regulation of ChIP-seq Target Genes *in vitro* and *in vivo* during Invasive Pulmonary Aspergillosis. (A). Enrichment of RNA-seq differentially expressed genes in GO/Funcat categories of up- and down-regulated genes in srbA cells under hypoxia 120 minutes versus WT (B). Analysis of transcript levels of 12 of the ChiP-seq target genes *in vivo* in a murine model of invasive pulmonary aspergillosis for wild type (CEA10) and *in vitro* under normoxic/hypoxic conditions for Δ*srbA* and wild type *in vivo* samples were at 48–96 hours post-infection (grey, n = 16). *in vitro* samples were wild type normoxia (red, n = 2) and hypoxia (blue, n = 6) followed by Δ*srbA* under normoxia (red, n = 2) and hypoxia (blue, n = 6). Time under hypoxia for both wild type and Δ*srbA* ranged from 30 to 120 minutes. Expression values are represented as total number of normalized counts per transcript. Quantitation and normalization was as follows: Digital counts for 60 genes (ChIP targets, housekeeping genes and other genes of interest) were adjusted for binding efficiency with background subtraction using the included positive and negative controls from the manufacturer as per NanoString nCounter data analysis guidelines. Data sets were normalized to facilitate across sample comparisons using the geometric mean of 20 stably expressed genes.

SrbA's role in hypoxic adaptation becomes evident at 120 minutes post-exposure to hypoxia with 520 genes having a 4-fold or greater decrease in transcript levels in the absence of SrbA compared to wild type. These genes are enriched for fatty acid and lipid biosynthesis, iron ion binding, carbohydrate metabolism, virulence, isoprenoid metabolism, and cellular oxidoreductase activity (Figure 2A). Importantly, the majority of SrbA direct target genes identified in our ChIP-seq analysis fall into this group of genes with reduced mRNA levels strongly suggesting that SrbA directly positively regulates their mRNA abundance in response to hypoxia. Conversely, 467 genes had a 4-fold or greater increase in transcript levels in the absence of SrbA compared to wild type. These genes were enriched in part for transcription factor activity, and as no enrichment of the SRE motif could be found in this dataset (P-value - 0.96 Fisher Exact test), we interpret the increase in transcript levels of these genes as indirect responses to the loss of SrbA activity in the cell, rather than SrbA acting as a transcriptional repressor of these genes. Alternatively, SrbA may positively regulate an unidentified transcriptional repressor(s). Taken together, these results strongly suggest that SrbA is a direct positive transcriptional regulator of genes required for adaptation to hypoxia. Moreover, these results strongly suggest that loss of SrbA fundamentally alters fungal cellular bioenergetics in response to hypoxia.

### Nanostring nCounter Transcript Abundance Analysis Reveals High Transcript Levels for SrbA Target Genes in a Murine Model of Invasive Pulmonary Aspergillosis

To gain insights into SrbA target genes critical for pathogenesis, we interrogated the mRNA abundance of a sub-set of SrbA target genes in an *in vivo* murine model of IPA, and *in vitro* in response to normoxia and hypoxia utilizing Nanostring nCounter technology [52], [53]. Consistent with the RNA-seq data, nCounter analyses confirmed that transcript abundance of sterol biosynthesis genes in hypoxia decreased *in vitro* with loss of *srbA* (Figure 2B). Moreover, *erg3B, erg11A, erg25A, erg25B*, all displayed high transcript levels *in vivo* in lung parenchyma tissue consistent with their SrbA-dependent hypoxia induction *in vitro* and the occurrence of hypoxia *in vivo* (Figure 2B, Table S5). Additional genes that showed strong SrbA dependency *in vitro* and high transcript levels *in vivo* included AFUB_091650 (*sit1*), a putative siderophore-iron transporter, that is critical for response to low iron and previously shown to be directly transcriptionally regulated by SrbA using ChIP-qPCR [38], [54]. Sit1 may be critical for virulence as mRNA abundance is higher *in vivo* than in either of the tested *in vitro* conditions. The high transcript levels of *sit1* early in infection confirm the iron-limited environment of the murine lung. These data support direct regulation of iron uptake *in vivo* by SrbA, and further support the link between SrbA, hypoxia adaptation, and iron homeostasis in *A. fumigatus*.

In addition to *sit1*, two other metabolic genes, *hem13* and *alcC*, show less SrbA dependency *in vitro* than the ergosterol biosynthesis genes, but significant transcript induction *in vivo*. Hem13 is a coproporphyrinogen III oxidase that involved in heme biosynthesis, and heme has been characterized as a major oxygen-sensing molecule in *S. cerevisiae* [55]. It has been reported that expression of genes encoding the main heme biosynthesis enzymes are induced in response to hypoxia in *S. pombe*, *C. neoformans*, and *A. fumigatus* [19], [27], [38]. In yeast, heme and oxygen negatively regulate HEM13 expression, and the repression of HEM13 involves a negative transcriptional regulator of hypoxic genes, ROX1 [56], [57]. AlcC is the alcohol dehydrogenase critical for ethanol fermentation in *A. fumigatus* and important for *in vivo* fungal growth as previously observed by Grahl *et al.* 2011. While SrbA is involved in regulation of these two metabolic genes, their dependency on SrbA is not as significant as genes involved in ergosterol biosynthesis.

**Figure 3:**
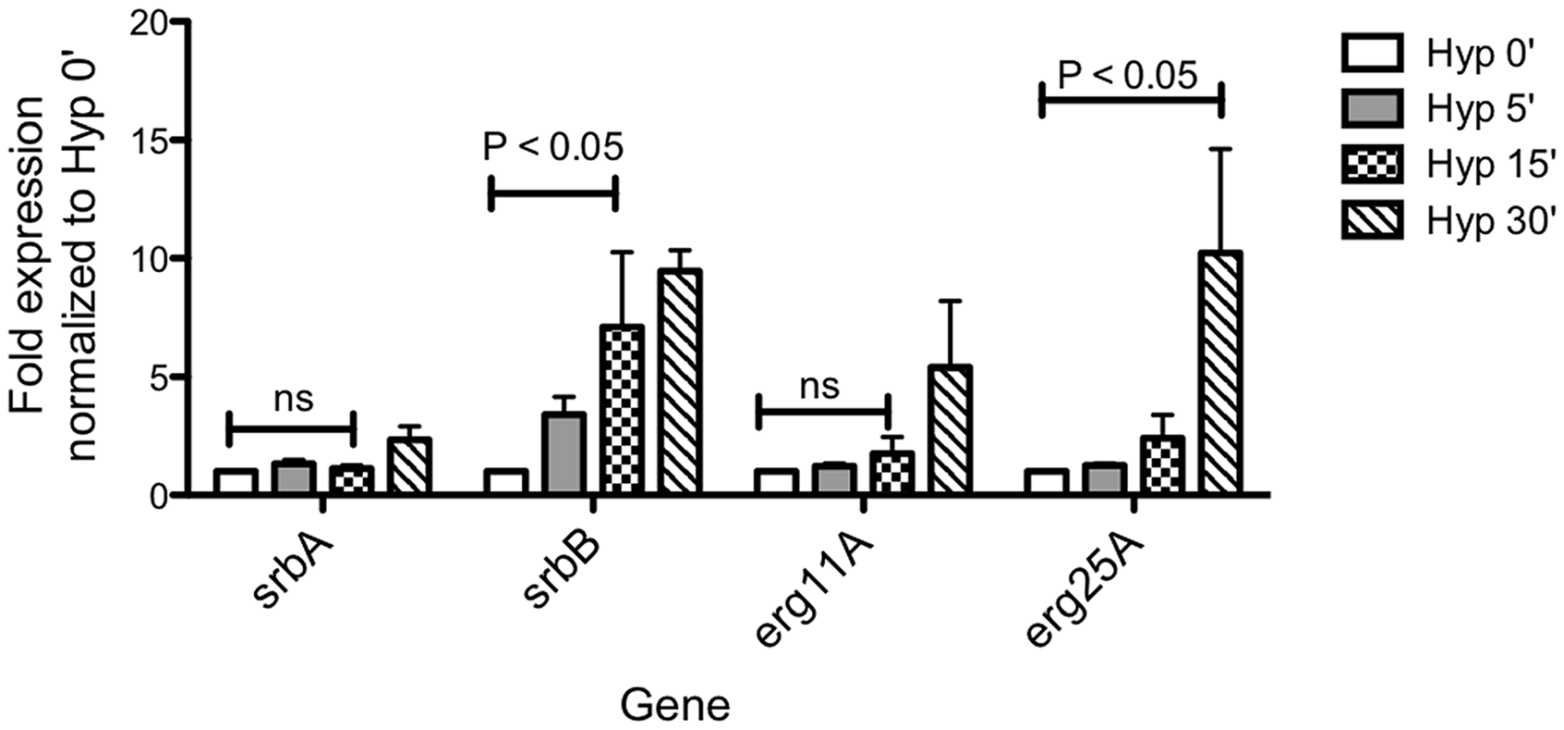
Temporal transcriptional induction of SrbA and SrbB in response to Hypoxia Reveals SrbB transcript levels are induced prior to SrbA. *Aspergillus fumigatus* wild type was cultured in normoxia at 37°C for 18 hours and shifted to hypoxia for additional incubation for 5, 15, and 30 minutes. Data are presented as the mean and standard error of three biological replicates. Compared to normoxia (Hyp 0′), *srbB* expression was significantly induced in hypoxia 15 min, which was earlier than the initial *srbA* induction. The data were analyzed by two-way ANOVA followed by Bonferroni posttests.

SrbA itself intriguingly displays higher transcript levels *in vivo* than *in vitro* and 2 additional putative transcriptional regulators, AFUB_090280 (Table S5) and AFUB_099590 (Figure 2B), display strong *in vitro* hypoxia induction that is partially SrbA dependent with strong expression *in vivo*. Previous informatics based analyses of AFUB_099590 [21], [58], suggested an important role for this DNA binding protein in both metabolism and virulence. Transcript levels for AFUB_099590, which we designate *srbB*, are highly induced in response to hypoxia *in vitro* in a partially SrbA dependent manner as our microarray based analysis of Δ*srbA* also previously suggested. Intriguingly, *srbB* transcript is one of the highest *in vivo* abundant genes relative to *in vitro* normoxic conditions. Moreover, a temporal analysis of *srbA* and *srbB* transcript levels in response to hypoxia revealed that *srbB* mRNA levels increase before *srbA* transcript levels and rise five minutes after exposure to hypoxia (Figure 3). Consequently, functional characterization of SrbB was undertaken with genetic and phenotypic analyses.

### Characterization of SrbB Reveals a New Hypoxia Transcriptional Regulator with a Role in *A. fumigatus* Virulence

Amino acid sequence alignment of SrbB (HLH domain, Figure S1) revealed that this protein is an SREBP homolog, as previously suggested [21]. Characteristic of SREBP family members, SrbB contains a tyrosine substitution in the basic portion of the bHLH domain. Intriguingly, SrbB does not contain any predicted transmembrane domains, suggesting this SREBP family member may not be regulated via proteolytic cleavage like SrbA.

**Figure 4:**
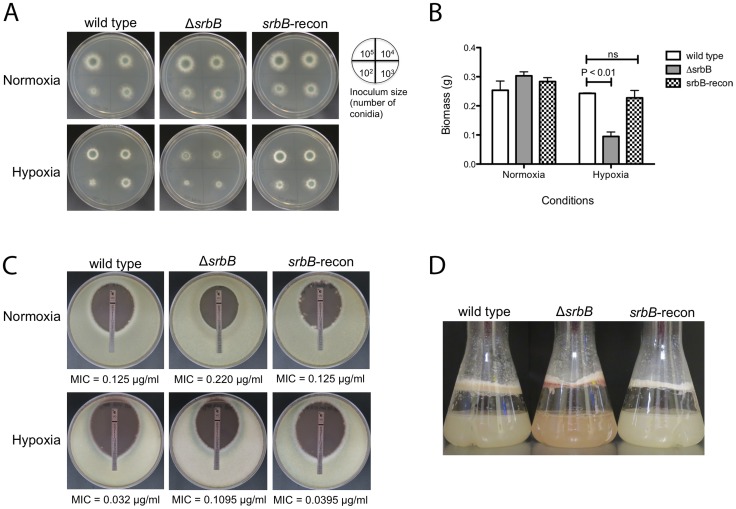
Loss of the Hypoxia Induced Transcriptional Regulator SrbB Results in a Significant Growth Defect and Red Pigmented Mycelia in Hypoxia. (A). Growth of Δ*srbB* in normoxia and hypoxia on solid media. Wild type, Δ*srbB*, and *srbB*-reconstituted strains were incubated on GMM at 37°C for 3 days in normoxia or hypoxia. The number of conidia used for inoculation is illustrated by the plate image. Compared to wild type and the reconstituted strain, growth of Δ*srbB* is restricted in hypoxia. (B). A biomass test with wild type, an *srbB* null mutant, and an *srbB* reconstituted strain in liquid cultures in normoxia or hypoxia. Mycelia of wild type, Δ*srbB* and *srbB*-reconstituted (*srbB*-recon) strains in liquid cultures were harvested, dried and weighed for the biomass study. Data are presented as the mean and standard error of three biological replicates. No significant differences between wild type and *srbB*-recon biomass were observed in all conditions tested. When analyzed by two-way ANOVA followed by Bonferroni posttest, biomass of Δ*srbB* was not different from wild type or *srbB*-recon in normoxia. However, biomass of Δ*srbB* significantly decreases in hypoxia compared to wild type (p<0.001). (C). E-test strips were utilized to test susceptibility to VCZ. 10^5^ conidia were overlaid on RPMI media, cultured at 37°C for 2 days. Minimal inhibitory concentrations (MIC, marked as an arrow) were measured. In both normoxia and hypoxia, Δ*srbB* is slightly more tolerant to VCZ compared to wild type and the *srbB* reconstituted strain. MIC ratios of Δ*srbB* to wild type are 1.76 and 3.42 in normoxia and hypoxia, respectively. (D). Conidia of each strain were cultured in LGMM at 37°C, 200 rpm for 2 days in hypoxia. Δ*srbB* produces reddish mycelia compared to the wild type and reconstituted strain.

Functional analysis of *srbB* was initiated through generation of a genetic null mutant and reconstituted strain (Figure S2). Loss of SrbB results in ∼50% reduction in colony radial growth on solid glucose minimal media (GMM) in hypoxia (Figure 4A). In addition to the decrease in growth rate on solid medium, Δ*srbB* colonies were noticeably less dense and often contained fluffy mycelia in hypoxic conditions. Though not statistically significant, there was a trend toward increased growth and mycelial density in normoxic conditions with Δ*srbB*. The impact of SrbB loss in response to hypoxia was pronounced in liquid GMM culture conditions where a significant reduction of biomass was observed compared to normoxia cultures (Figure 4B). These results strongly suggest that SrbB is a major transcriptional regulator of the fungal response to hypoxia. Unlike our previous reports with Δ*srbA*, a minimal increase in tolerance to the triazole voriconazole was observed in Δ*srbB* in normoxia and hypoxia suggesting a limited role for SrbB in regulating responses to triazoles (Figure 4C). Of significant note, upon culture in liquid GMM, Δ*srbB* exhibited a strong red pigmentation of the mycelia (Figure 4D). This coloration was limited to the mycelia, and only occurred in hypoxic conditions.

### SrbB is a Major Hypoxia Transcriptional Regulator of Carbohydrate, Heme, and Lipid Metabolism

**Figure 5:**
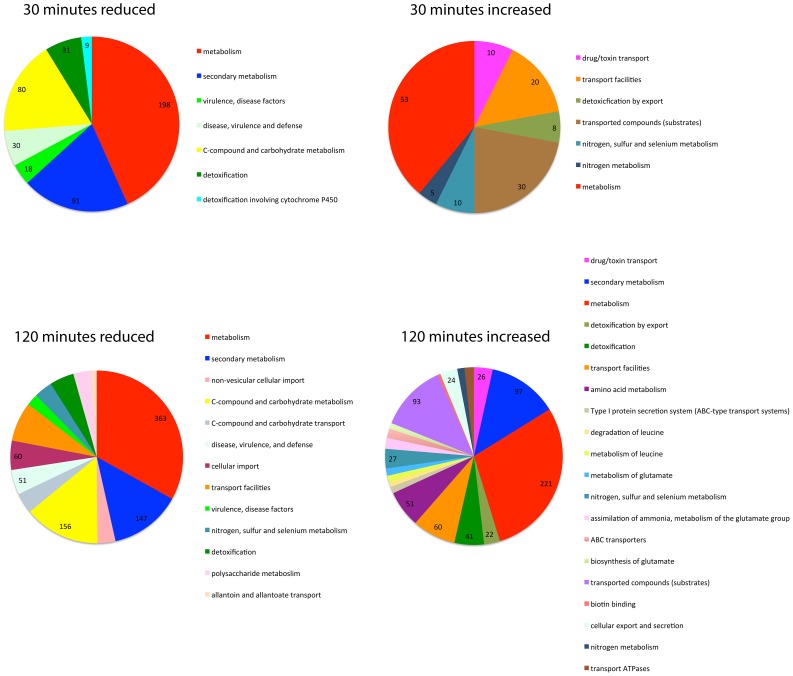
SrbB is a transcriptional regulator of genes involved in carbon metabolism, lipid metabolism, and heme biosynthesis. The FungiFun2 web server was utilized to assign FunCat and gene ontology enrichment in genes with transcript levels increased or decreased 4 fold in Δ*srbB* compared to the wild type strain. Statistically significant (P≤0.05) FunCat categories are presented at the respective time points in hypoxia.

To better understand SrbB's function in hypoxia, the origin of the red pigment produced in Δ*srbB* hypoxic mycelia, and SrbB's genetic relationship to SrbA, RNA-seq analysis was conducted with Δ*srbB* and wild type under the same conditions and time points as Δ*srbA* (Figure 2, Figure 5). In contrast to Δ*srbA*, but consistent with SrbB's early induction in response to hypoxia (Figure 3), the transcriptome of Δ*srbB* was significantly changed at 30 minutes post-exposure to hypoxia. 490 genes had 4 fold or greater reductions in transcript levels in Δ*srbB* compared to wild type under hypoxic conditions (Table S4 and S6). These genes were enriched (P≤0.05) in FunCat categories of carbohydrate metabolism, virulence, secondary metabolism, and detoxification (Figure 5). In contrast, 135 genes had transcript levels increase 4-fold or greater in Δ*srbB* compared to wild type at this early hypoxia time point. Genes with increased transcript levels in Δ*srbB* were enriched in transport of toxic products, which could suggest a global dysregulation of metabolism in Δ*srbB*. At 120 minutes post-exposure to hypoxia, the impact of SrbB loss on transcript levels was particularly strong, with 1026 transcripts decreasing 4-fold or greater. Over half of these genes are not currently annotated (522), but those annotated are enriched in carbohydrate and nitrogen metabolism, virulence, secondary metabolism, and transport of various molecules. Moreover, at 120 minutes 491 genes had transcript levels increased 4-fold or greater in Δ*srbB* compared to wild type. Perhaps associated with the enrichment of toxic product detoxification in the genes with reduced transcript levels, genes with increased transcript levels in Δ*srbB* were associated strongly with amino acid metabolism and degradation. Overall, these data suggest a significant metabolic or bioenergetics dysregulation in *A. fumigatus* cultured under hypoxic conditions when SrbB function is absent. Consequently, loss of SrbB results in major alterations of the *A. fumigatus* hypoxia transcriptome that impact hypoxia growth.

**Figure 6:**
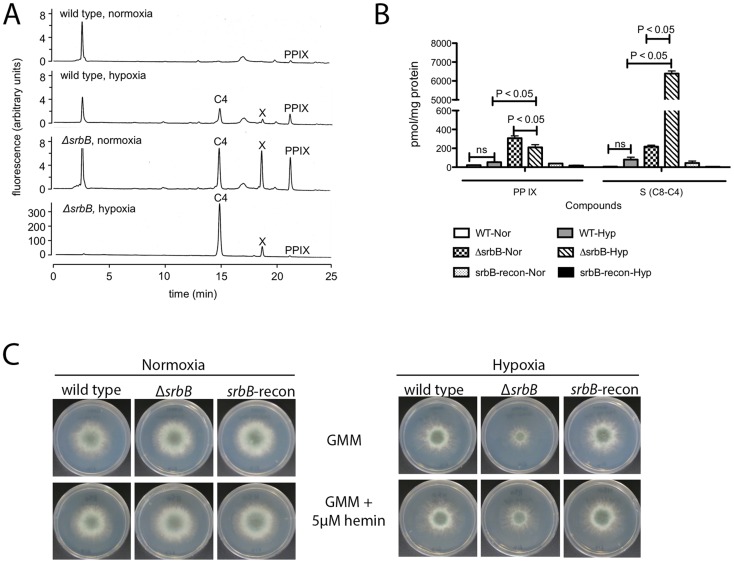
Loss of *srbB* impairs heme biosynthesis and results in accumulation of heme intermediates. (A–B). Amount of Protoporphyrin IX (PP IX) and intermediate compounds including uroporphyrin (C8), heptacarboxylporphyrin (C7), hexacarboxylporphyrin (C6), pentacarboxylporphyrin (C5), and coproporphyrin (C4) were analyzed using HPLC. Mycelia used for HPLC analysis were harvested from cultures in LGMM at 37°C for 2 days in hypoxia. Compared to wild type, Δ*srbB* produces more PP IX and other intermediates in hypoxia. (A) is a chromatogram from HPLC analysis, and (B) is a graph to present the HPLC result with statistical analysis. Data are presented as the mean and standard error of three biological replicates, and analyzed by one-way ANOVA followed by a Tukey's multiple comparison test. (C). A thousand conidia were inoculated on GMM or GMM containing 5 µM hemin. In 2 days, radial growth of each strain in normoxia or hypoxia was observed. Addition of hemin improved Δ*srbB* growth in hypoxia.

Interestingly, *hem13* transcript levels were markedly reduced in Δ*srbB* mycelia exposed to hypoxia, providing a potential cause of the red pigment previously observed in this strain. To test the hypothesis that the red pigmentation of Δ*srbB* is associated with a defect in heme metabolism, we quantified the accumulation of heme biosynthesis intermediates in Δ*srbB* in normoxia and hypoxia (Figure 6A–B). Consistent with the red pigmentation and RNA-seq analysis of Δ*srbB*, a striking accumulation of heme biosynthesis intermediates including protoporphyrin IX were observed in Δ*srbB* (Figure 6A–B). Intriguingly, when 5 µM hemin was exogenously added into the culture media, Δ*srbB* growth was significantly improved in hypoxia compared to GMM alone (Figure 6C). In contrast, addition of hemin to Δ*srbA* did not restore this strain's growth under the conditions examined. Taken together, these data suggest that SrbB is a critical regulator of heme biosynthesis in *A. fumigatus* and that accumulation of toxic heme intermediates may contribute to the hypoxia growth defect observed in Δ*srbB*.

### SrbA and SrbB have Dependent and Independent Functions in Hypoxic Gene Regulation

**Figure 7:**
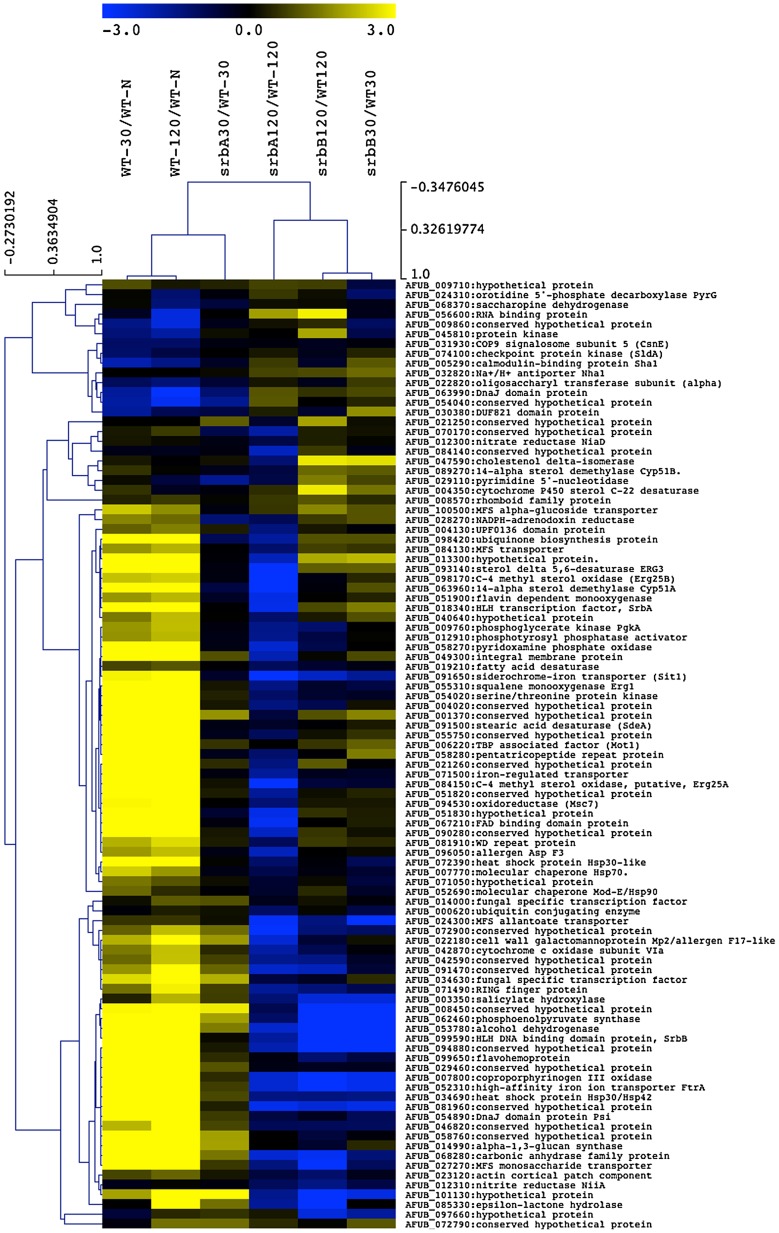
A sub-set of SrbA ChIP Target Genes are co-regulated by SrbB. The RNA-seq data for annotated genes corresponding to SrbA ChIP-seq peaks are shown as ratios of 30 and 120-minute wild-type hypoxia vs. wild-type normoxia, and gene deletion strains are shown as the deletion strain vs. the equivalent wild type hypoxia time point. Genes discussed and/or examined in detail in this manuscript are noted with asterisks. MeV analysis was performed using hierarchical clustering. Optimized gene and leaf ordering groups the wild type 30- and 120-minute hypoxic conditions together, with the 30-minute Δ*srbA* sample more similar to wild type for the SrbA targets, using Pearson correlation with complete linkage clustering.

Inspection of genes regulated by SrbA and SrbB identified using ChIP-seq and RNA-seq analyses suggested a close functional relationship between these two SREBP family members. To compare expression patterns of strains lacking each respective transcription factor, a hierarchical clustered heat map was generated for SrbA direct annotated target genes (Figure 7). Examination of the heat map reveals that a sub-set of SrbA target genes also depends on SrbB for wild-type transcript levels in hypoxia. Examples of these target genes include *hem13, niiA*, and *erg25A*. In contrast, a sub-set of SrbA target genes had increased or wild type transcript levels in the absence of SrbB. These include *srbA* itself and its target genes *erg11A/cyp51A, erg11B/cyp51B*, and *erg3B*. These latter genes appear to be dependent on SrbA and not SrbB under the examined conditions.

**Figure 8:**
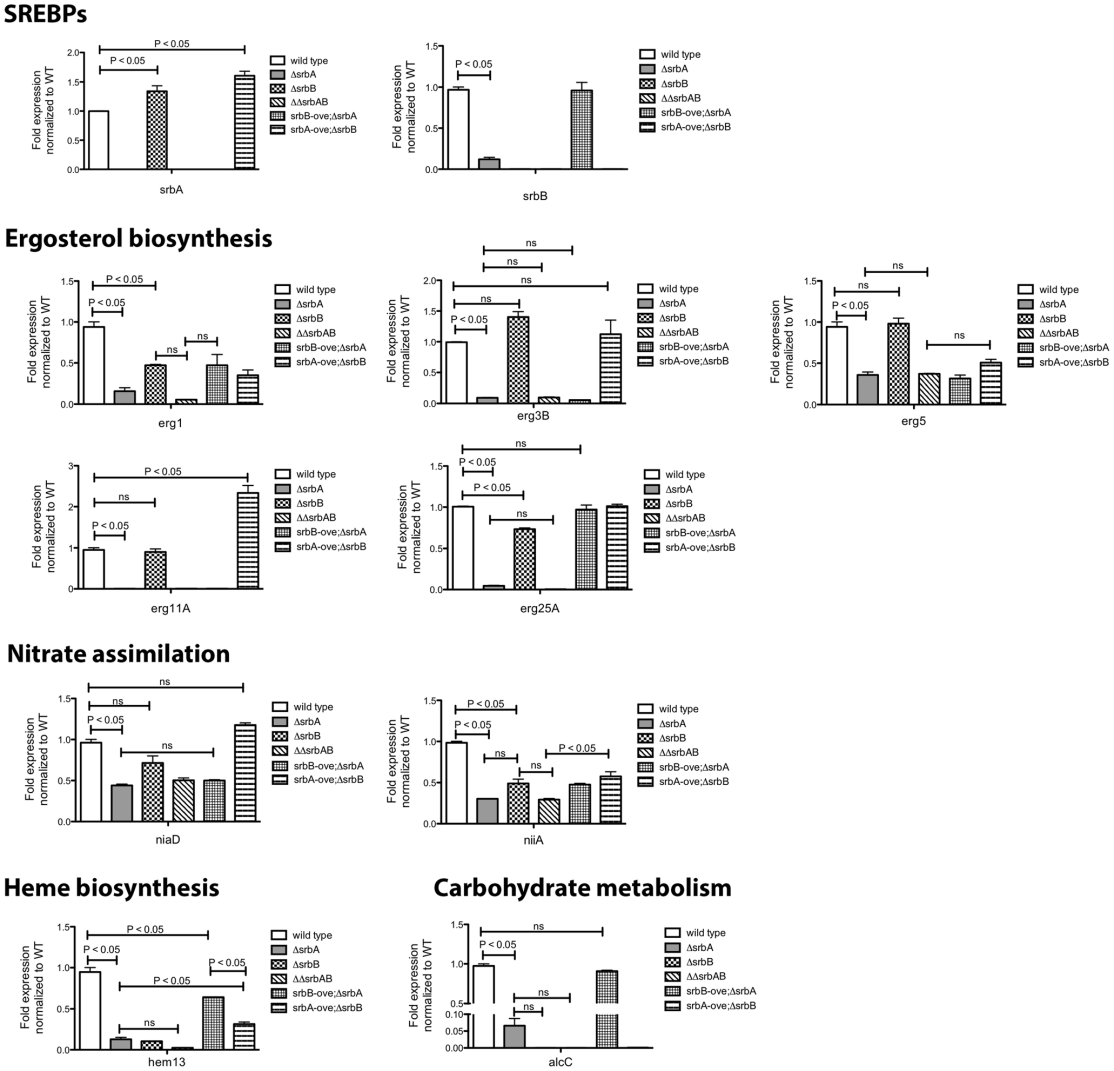
Co-Regulation of SrbA target genes by SrbB. Conidia of each strain were cultured in normoxia at 37°C, 250 rpm for 18 hours and shifted to hypoxia for additional incubation for 4 hours. Expression of SrbA target genes involved in ergosterol biosynthesis, nitrate assimilation, heme biosynthesis, and carbohydrate metabolism in the strains was studied using qRT-PCR. Data are presented as the mean and standard error of two biological replicates, and analyzed by one-way ANOVA followed by Bonferroni's posttests. Expression of *erg1*, *erg25A*, *niiA*, *hem13*, and *alcC* requires both SrbA and SrbB. In contrast, expression of *erg3B*, *erg11A*, and *niaD* are regulated by only SrbA. SrbB appears to have a dominant role over SrbA in regulation of *hem13* and *alcC* expression. ΔΔ*srbAsrbB*: a double knock-out mutant of *srbA* and *srbB*, *srbB*-ove;Δ*srbA*: Δ*srbA* with restored *srbB* expression, *srbA*-ove;Δ*srbB*: a *srbA* overexpression strain in Δ*srbA*Δ*srbB*.

Next, to further define the genetic relationship between SrbA and SrbB, we generated 3 additional strains: (1) Δ*srbA*Δ*srbB* (2) *srbB* over-expression in Δ*srbA* (*srbB*-ove;Δ*srbA*) and (3) *srbA* over-expression in Δ*srbA*Δ*srbB* (*srbA*-ove;*ΔsrbB*). In order to over-express *srbB* in Δ*srbA*, the *A. fumigatus* flavohemoprotein (*flavA*(p), AFUB_099650) or *A. nidulans* glyceraldehyde 3-phosphate dehydrogenase (*gpdA*(p), AN8041) promoters were utilized. These promoters were chosen because their respective genes are highly expressed in hypoxia in glucose minimal media with nitrate as a sole nitrogen source. The *flavA*(p) was chosen from the RNA-seq conditions where AFUB_099650 was one of the highest expressed genes in response to hypoxia [59]. Previously, in *A. oryzae*, it was reported that the FlavA homolog responds strongly to nitric oxide stress, which could occur in our media conditions with NO_3_ as a nitrogen source [60]. The resulting strains were designated TDC43.18 (*flavA*(p):*srbB*;Δ*srbA*) and TDC44.2 (*gpdA*(p):*srbB*;Δ*srbB*), respectively, and TDC43.18 was selected to study gene expression in the *srbB*-ove;Δ*srbA* strain. qRT-PCR analysis of select SREBP targets was conducted on cultures exposed to hypoxia for four hours, similar to ChIP-seq conditions. Consistent with the RNA-seq data, loss of SrbB resulted in a modest increase in *srbA* transcript levels (Figure 8). Conversely, loss of SrbA results in a strong decrease in *srbB* transcript levels suggesting that SrbA positively regulates *srbB* mRNA levels in hypoxia.

Transcript levels of nine genes, plus *srbA* and *srbB*, representing ergosterol biosynthesis, heme biosynthesis, carbohydrate metabolism, and nitrate assimilation from ChIP-seq data were investigated in Δ*srbA*, Δ*srbB*, Δ*srbA*Δ*srbB*, *srbB*-ove;Δ*srbA*, and *srb*A-ove;Δ*srbB* compared to wild type in hypoxia for four hours. Genes that require both SrbA and SrbB for full abundance include *erg1, erg25A, niiA*, and *hem13* as suggested by the RNA-seq analysis. In contrast, expression of *erg3B*, *erg5*, *erg11A*, and *niaD* requires SrbA but not SrbB (Figure 8). However, interpretation of this data is complicated by the fact that SrbA positively regulates *srbB* transcript levels. This is exemplified with mRNA levels of the ethanol fermentation and virulence factor *alcC*. Loss of SrbA significantly reduces *alcC* transcript levels, but not to the extent as loss of SrbB (Figure 8). SrbA is modestly enriched on the *alcC* promoter (Table 1, Table S2, Figure 1). However, over-expression of SrbB in Δ*srbA* essentially fully restores *alcC* transcript levels. Taken together, these results strongly suggests that *alcC* transcript levels are primarily regulated by SrbB, although SrbA can bind the SRE site found in the *alcC* promoter and contribute to its regulation.

**Figure 9:**
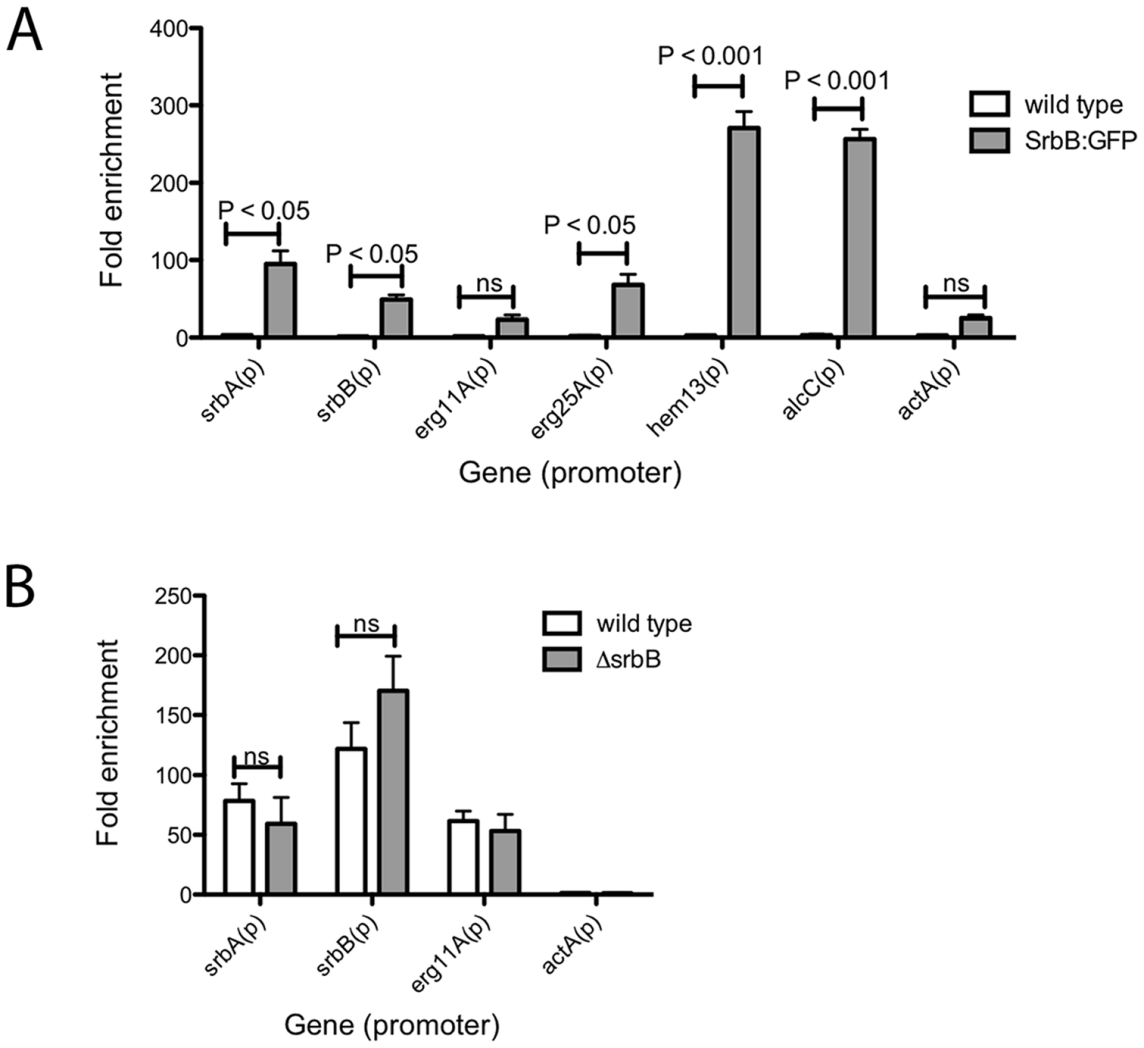
Binding of SrbB to the promoter of specific SrbA target genes and binding of SrbA in Δ*srbB*. (A) SrbB tagged with GFP was expressed in *A. fumigatus* wild type. The resulting strain was cultured in normoxia at 37°C, 250 rpm for 18 hours and shifted to hypoxia for additional incubation for 4 hours. ChIP was conducted using GFP antibody followed by ChIP-qPCR to study SrbB enrichment on the promoters of SrbA target genes. Compared to wild type control, SrbB enrichment was significant in SrbB:GFP for *srbA*, *srbB*, *erg25A*, *hem13*, and *alcC*, which suggest SrbB directly binds on the promoter of these genes for transcriptional regulation. In contrast, SrbB enrichment on the promoters of *erg11A* and *actA* were not significant. Data are presented as the mean and standard error of two biological replicates, and analyzed by two-way ANOVA followed by Bonferroni posttest. (B) SrbA binding to the promoter of *srbA*, *srbB*, and *erg11A* in Δ*srbB* was examined by ChIP-qPCR. Compared to wild type, SrbA enrichment on the gene promoters was not altered by disruption of SrbB. Data are presented as the mean and standard error of two biological replicates and analyzed by two-way ANOVA followed by Bonferroni posttests.

To seek further insights into regulation of a sub-set of these target genes by SrbA and SrbB, we performed ChIP-qPCR with a SrbB:GFP strain using a GFP antibody. SrbB tagged with GFP was ectopically expressed in the *A. fumigatus* wild type background. Transcript levels of *srbB* in the SrbB:GFP strain under the same conditions used for ChIP-qPCR was similar to wild type, and SrbB:GFP is localized to the nucleus in this strain (Figure S3). Cultures for ChIP were prepared in hypoxia for 4 hours and enrichment of SrbB on the SrbA binding sites of *srbA*, *srbB*, *erg11A*, *erg25A*, *hem13* and *alcC* was examined compared to wild type (Figure 9A). The negligible SrbB enrichment on the actin promoter supports binding specificity of the GFP antibody used for ChIP. ChIP-qPCR results show that SrbB binds to the SrbA binding sites of *srbA*, *erg25A*, and *hem13* whose transcript levels require both SrbA and SrbB as described above.

In contrast, SrbB enrichment on the SrbA binding site of *erg11A*, whose transcript level solely relies on SrbA, was not significant compared to wild type. Interestingly, similar to SrbA, SrbB binds to its own promoter (Figure 9A). We next tested whether loss of SrbB would affect binding of SrbA to its direct target genes (Figure 9B). Compared to wild type, SrbA binding on the promoters of *srbA*, *srbB*, and *erg11A* did not change in Δ*srbB*. Considering that both SrbA and SrbB are able to bind promoters of hypoxic genes including *srbA*, *srbB*, and *erg11A* (Figure 9A), these two transcription factors may form either homodimers or heterodimers to regulate gene expression. Thus, similar SrbA enrichment between wild type and Δ*srbB* observed in Figure 9B is likely because the SrbA homodimer (or monomer) is the major form bound to the promoters of these genes. Overall, our data suggest that SrbA and SrbB are critical for hypoxia adaptation in *A. fumigatus* with both dependent and independent functions in regulation of genes critical for hypoxia adaptation and growth.

### Restoration of Full *srbB* Transcript Levels in the Δ*srbA* Background Partially Restores Hypoxic Growth

**Figure 10:**
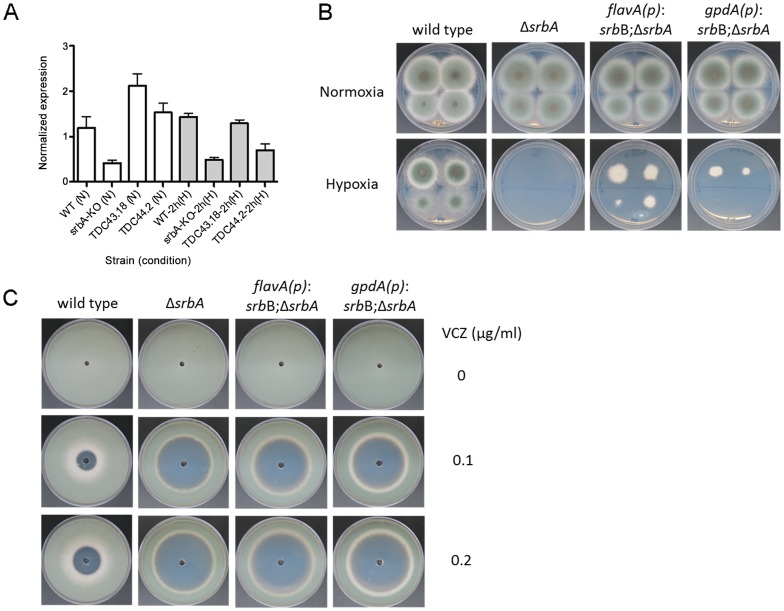
Restoration of *srbB* transcript levels in Δ*srbA* promotes growth in hypoxia. (A). Using two promoters, *flavA*(p) and *gpdA*(p), *srbB* was expressed in Δ*srbA*. Expression levels of *srbB* in TDC43.18 (*flavA*(p)) and TDC44.2 (*gpdA*(p)) in normoxia or hypoxia 2 h were verified using quantitative PCR. Data are presented as the mean and standard error. Compared to Δ*srbA*, *srbB* transcript in TDC43.18 and TDC44.2 was more abundant by 5- and 3.7-fold in normoxia and 2.7- and 1.4-fold in hypoxia 2 h, respectively. This indicates these promoters function properly. (B). Sequentially diluted conidia (10^2^–10^5^) were inoculated on GMM plates and culture in normoxia or hypoxia at 37°C for 3 days. Increased expression of *srbB* using *flavA*(p) or *gpdA*(p) partially restores defective hypoxia growth in Δ*srbA*. (C). Susceptibility of TDC43.18 and TDC44.2 to the triazole drug, voriconazole (VCZ) was tested. A million conidia were overlaid on GMM, and VCZ in DMSO was applied to the center of the plate. Cleared areas represent inhibited fungal growth in response to VCZ. Although growth in hypoxia was partially rescued in both strains as shown (B), increased susceptibility to VCZ in Δ*srbA* compared to wild type was not affected by restoration of *srbB* expression.

These data suggest that SrbB co-regulates a sub-set of SrbA target genes and that loss of SrbA also markedly reduces SrbB levels. Consequently, we hypothesized that restoration of full SrbB levels in Δ*srbA* may ameliorate the severe hypoxic growth defect of Δ*srbA*. As described above, we generated two strains that have restored *srbB* expression in Δ*srbA* using two promoters, *flavA(p)* and *gpdA(p)* (TDC43.18 and TDC44.2, respectively). Quantitative real-time PCR was conducted to verify if these promoters induced transcript level increases of *srbB*. Compared to Δ*srbA*, *srbB* transcript levels increased by 5.1- and 3.7-fold in normoxia and 2.7- and 1.4-fold in hypoxia in TDC43.18 (Figure 10A). Restoration of *srbB* transcript levels strongly promotes growth of Δ*srbA* in hypoxia, however growth is not fully restored to wild type levels and was dependent on *srbB* transcript levels (Figure 10B). In addition, as predicted, restoration of *srbB* transcript levels in Δ*srbA* did not rescue the triazole drug susceptibility of Δ*srbA* (Figure 10C). This result is consistent with *cyp51A/erg11A* mRNA levels primarily regulated directly by SrbA.

The Δ*srbA*Δ*srbB* strain generated to study gene transcript levels of SrbA target genes was further characterized to study the genetic relationship between SrbA and SrbB. Δ*srbA*Δ*srbB* showed similar phenotypes to Δ*srbA*, with a complete lack of growth in hypoxia and marked increased in azole drug susceptibility compared to wild type (Figure S4A–B). Moreover, when *srbA* was over-expressed in Δ*srbA*Δ*srbB*, the resulting strain grew similar to wild type in hypoxia (Figure S4C). Taken together, these data are consistent with a model where SrbA positively regulates *srbB* gene expression in response to hypoxia. It also further supports that both SrbA and SrbB are involved in hypoxic gene regulation but SrbA plays a dominant role over SrbB with regard to genes essential for hypoxia growth in the tested conditions.

### SrbB is Required for Full Virulence of *Aspergillus fumigatus*

**Figure 11:**
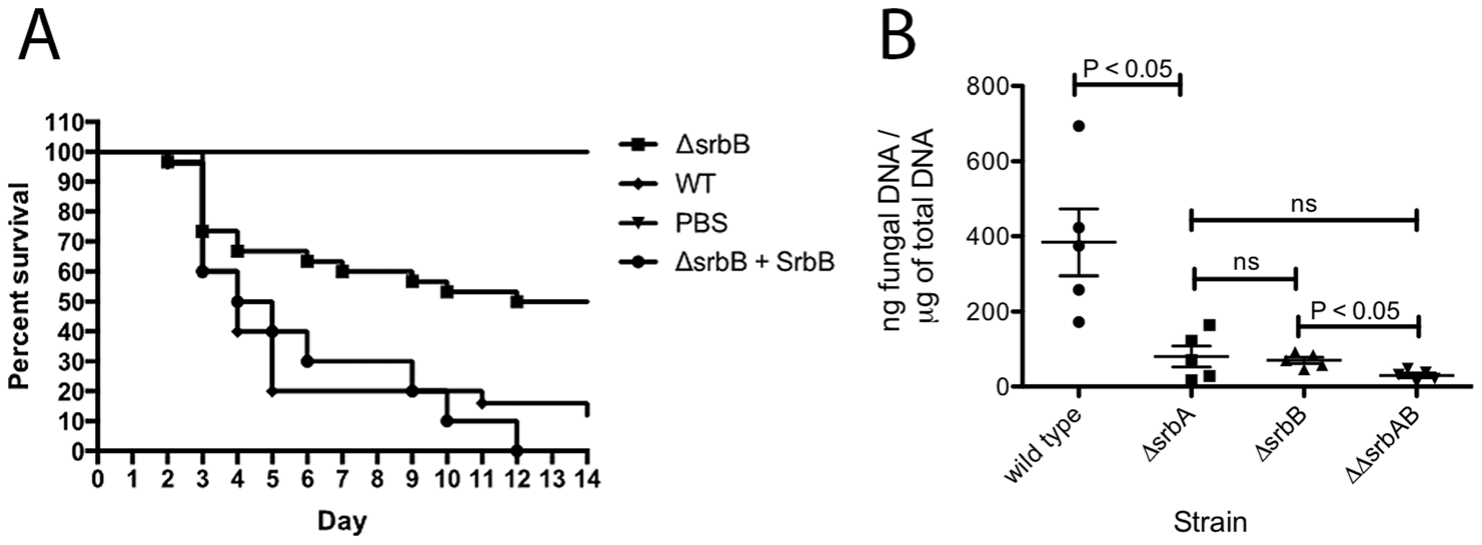
Loss of SrbB attenuates *Aspergillus fumigatus* virulence through reductions in pulmonary fungal burden. (A). 6–8 week old immunosuppressed CD-1 mice (each group N = 20) inoculated via the intranasal route with 2×10^6^ conidia of wild type, Δ*srbB* and *srbB*-reconstituted strains. Comparing wild type and reconstituted strain Kaplan-Meier curves with the Δ*srbB* strain shows a significant increase in survival for the animals inoculated with the Δ*srbB* strain (Log rank test, p = 0.0027). All control PBS inoculated animals survived. (B) Triamcinolone mouse model was used for fungal burden analysis. Mice were infected with 10^6^ conidia of each strain and lungs were collected on day +3. Data represented are the mean and standard error of 3–5 mice per group.

Of particular importance for understanding *A. fumigatus* pathogenesis, loss of SrbB results in a significant virulence attenuation in a steroid murine model of IPA (Figure 11A). Kaplan-Meier curves with Δ*srbB* show a significant increase in survival for animals inoculated with Δ*srbB* compared to wild type and reconstituted strains (Log rank test, p = 0.0027). To gain insights into the relative contributions of SrbA and SrbB to virulence, we examined total fungal growth *in vivo* utilizing qRT-PCR based quantitation of fungal burden as we have previously described (Figure 11B) [7], [61]. As expected, Δ*srbA* displayed a significant decrease in fungal burden compared to wild type consistent with its attenuated virulence in murine models of IPA (P = 0.008) [36], [37]. Surprisingly, Δ*srbB* fungal burden was as low as Δ*srbA* despite it having a higher level of virulence than Δ*srbA* as measured by murine survival. Consequently, Δ*srbA*Δ*srbB* exhibited an even a greater reduction in virulence than either single mutant alone (P = 0.016 between Δ*srbB* and Δ*srbA*Δ*srbB*), though the difference with Δ*srbA* did not achieve statistical significance (P = 0.31), Figure 11B). Taken together, these data suggest that SrbB is an important SrbA-dependent regulator of the fungal response to hypoxia and required for full fungal virulence, and that both SrbA and SrbB make novel contributions to *A. fumigatus* virulence.

## Discussion

Previously, a link between the transcription factor SrbA, and the ability of *A. fumigatus* to cause disease in murine models of IPA has been observed [36]–[38]. However, the mechanism(s) by which SrbA mediates *in vivo* fungal growth and virulence are not fully defined. Given the conservation of SrbA with mammalian SREBPs, uncovering the fungal specific functions of SrbA is important in order to yield potential mechanisms that can be targeted for therapeutic development. Support for this rationale comes from several elegant studies showing that targets of conserved transcription factors are often not shared between distantly related organisms and even closely related species [62]–[65]. Thus, in order to fully maximize the attenuated virulence of fungal pathogens lacking SREBP homologs for therapeutic benefit, in-depth investigation into their regulons and mechanisms of regulation and activity are needed.

Here, the *A. fumigatus* SrbA transcriptional regulon in response to hypoxia was interrogated utilizing ChIP-seq, RNA-seq and Nanostring nCounter technologies. Ninety-seven genes whose promoters were strongly bound by SrbA were identified in the ChIP-seq analysis. This number appears low considering the large number of genes differentially expressed in the absence of SrbA as detected here via RNA-seq and previously with microarray analysis [38]. However, a low overlap between transcript levels of genes affected by transcription factor loss and target genes identified in ChIP experiments appears to be the norm rather than the exception [66]. Consequently, our results suggest that we may have herein underestimated our direct SrbA targets or, more likely, there is an extensive network of genes involved with SrbA that are indirectly regulated though other transcription factors, small molecules, and proteins. The identification of ergosterol biosynthesis genes as SrbA direct targets and the sequence conservation of the ChIP identified SRE DNA binding motif strongly support the robustness of our analyses.

A major novel finding from our study of SrbA target genes was the identification of SrbB and its genetic relationship with SrbA. Based on sequence similarity, Bien and Espenshade hypothesized that SrbB was an SREBP-like transcription factor [21]. While our amino acid sequence analysis of SrbB did not reveal the presence of transmembrane domains that are found in the prototypical SREBPs, SrbB contains the hallmark tyrosine residue in the bHLH domain characteristic of SREBP family members. Moreover, a *srbB::GFP* fusion protein was observed to localize solely to the nucleus on preliminary analyses (Figure S3) further suggesting that SrbB is not membrane bound like SrbA and other SREBP family members (though constitutive cleavage cannot currently be ruled out). Transcript levels of *srbB* were strongly induced in response to hypoxia, earlier and to a greater magnitude than *srbA*. Thus, transcriptional regulation of *srbB* mRNA levels appears to play a major role in regulation of its function. In hypoxia, SrbA was found to bind to the promoter region of *srbB* at three locations. Loss of SrbA, however, did not completely eliminate *srbB* transcript levels in hypoxia suggesting the presence of additional *srbB* transcriptional regulators that remain to be defined.

Consistent with increased transcript *in vitro* in response to hypoxia and strong induction of transcript levels *in vivo* in a murine model of IPA, loss of SrbB markedly attenuated hypoxia growth and virulence of *A. fumigatus*. Unlike loss of SrbA, loss of SrbB had a minor effect on susceptibility to triazole antifungal drugs. The lack of an azole drug phenotype is consistent with the observation that SrbB does not appear to regulate *cyp51A/erg11A* mRNA levels, at least under the conditions tested here. However, full expression of the ergosterol biosynthesis genes *erg1* and *erg25A* does require the presence of SrbB. In a similar manner, a major finding with regard to SrbB function is its clear importance for regulation of heme biosynthesis in normoxia and hypoxia. Heme plays a critical role in oxygen sensing and gene expression regulation in many organisms, and here our results link SrbB mediated regulation of heme biosynthesis with *A. fumigatus* hypoxia growth and virulence [67]–[70]. While SrbA binds to the promoter of *hem13*, and *hem13* mRNA levels are reduced in Δ*srbA*, SrbB appears to be the major transcriptional regulator required for *hem13* in hypoxia and consequently heme biosynthesis (Figure 8). Further investigations are needed into the role of heme in oxygen sensing and virulence in *A. fumigatus*.

SrbB displays significant sequence similarity with the recently characterized *Aspergillus oryzae* transcription factor *sclR* that is critical for hyphal morphology and sclerotial formation [71]. Intriguingly, the authors noted that loss of *sclR* negatively affected pellet formation in liquid culture. Δ*sclR* pellets exhibited hollow interiors and fluffy exteriors that is consistent with SclR having a role in hypoxic adaptation. In addition to *sclR*, SrbB has sequence similarity with the *Candida albicans* bHLH transcription factor Cph2, though reciprocal BLAST analyses do not confirm orthology. Like *srbB, CPH2* is expressed *in vivo* in models of candidiasis and is required for colonization of the murine gastrointestinal tract [72]–[75]. Its role in hypoxia adaptation in *C. albicans* is unclear; though also similar to SrbB it is induced early in the *Candida* hypoxic response [76].

**Figure 12:**
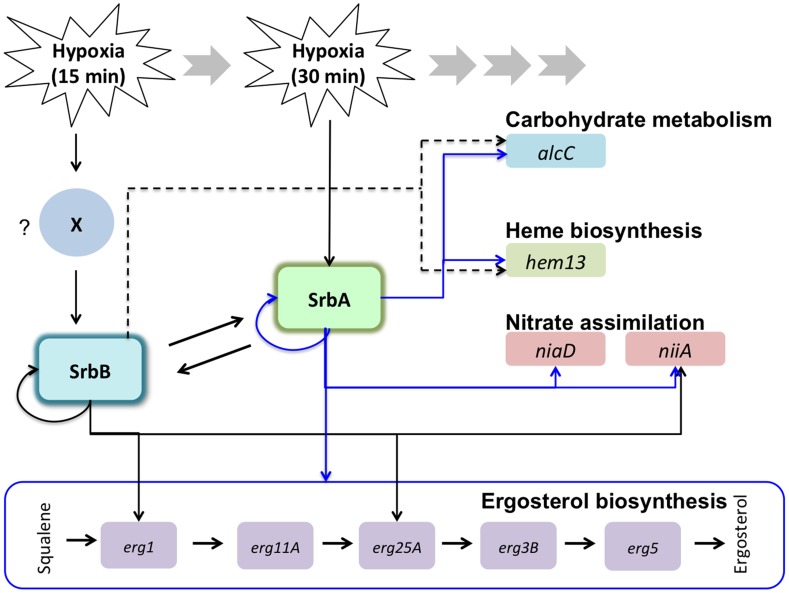
Working models for transcriptional regulation of the hypoxia response by SrbA and SrbB in *A. fumigatus*. *A. fumigatus* SREBPs, SrbA and SrbB have dependent and independent functions in regulation of hypoxic genes involved in ergosterol biosynthesis, carbohydrate metabolism, heme biosynthesis, and nitrate assimilation. In response to hypoxia, *srbB* transcription is induced earlier than *srbA* (15 minutes in hypoxia) possibly by an unknown factor (marked as ‘X’). SrbA regulates *srbB* and its own transcript abundance. Similarly, SrbB regulates *srbA* and its own transcript abundance. qRT-PCR and ChIP-qPCR data presented in this study show that abundance of *erg1*, *erg25A*, *hem13*, *niiA*, and *alcC* is regulated by both SrbA and SrbB. In contrast, abundance of *erg11A*, *erg3B*, *erg5*, and *niaD* is dependent on SrbA. SrbB appears to have a dominant function over SrbA in regulation of *hem13* and *alcC* transcript abundance (indicated by dotted lines).

While further analyses are needed to define the genetic and potentially physical relationships between SrbA and SrbB in *A. fumigatus*, our data hint at likely mechanisms that are modeled in Figure 12. First, data suggest that SrbA and SrbB have both mutually exclusive and co-regulated target genes in their regulons, and that they are involved in reciprocally regulating transcript levels. The increase in *srbA* transcript levels in the absence of *srbB* suggests that at some level SrbB could act as a transcriptional repressor. It is unclear if the effect on *srbA* transcript levels is direct and experiments to uncover direct SrbB target genes are ongoing in our laboratory. Analysis of SrbB DNA binding utilizing a SrbB:GFP fusion protein strongly suggests that SrbB can bind the SRE motif found in the *srbA* promoter region. It is likely, however, that the increase in transcript levels of several SrbA target genes in Δ*srbB* is driven by increases in SrbA levels and activity. In this model, besides its role as a transcriptional activator of genes important for the hypoxia response, SrbB also functions in a negative feedback loop to modulate SrbA activity. Accordingly, persistence of high SrbA levels would at some point become detrimental to cellular homeostasis perhaps through increases in an SrbA dependent molecule(s) that become toxic to the cell. We note that genes associated with export of toxic molecules were enriched in the upregulated gene set in Δ*srbB*.

Of course there are several possible mechanisms through which SrbA and SrbB could co-regulate gene transcription, however in mammals it is known that SREBPs can physically interact to regulate gene expression through formation of homo and heterodimers [77], [78]. It may be possible that genes co-regulated by SrbA and SrbB in response to hypoxia are regulated through heterodimer formation, while genes exclusive to the respective transcription factor are regulated in part by homo-dimer formation. Moreover, in mammals, SREBP homo-and heterodimers have different levels of transcriptional activity [77]. Thus, it is plausible that in Δ*srbB*, SrbA homodimers are more potent activators of SrbA target genes such as *srbA* itself than an SrbA-SrbB heterodimer that is present in the wild type. It is well established that the ability of bHLH proteins to form multiple dimer combination based on availability of binding partners and environmental conditions is a critical and elegant form of gene regulation [79]. Along these lines, genetic null mutants of fungal SREBPs alter the stoichiometric ratios of potential binding partners, which is further complicated by the fact that SrbA and SrbB are involved in regulating the other's expression. We also note that a third SREBP family member, SrbC, exists in *A. fumigatus* and awaits further investigation. Whether a preferred dimer form exists for regulation of specific SREBP regulated genes has not been extensively studied. Together, our data suggest that transcriptional activity of SrbA homodimer is efficient to activate *erg3B*, *erg5*, *erg11A*, and *niaD*, and an SrbA homodimer might be a preferred or dominant dimerization form for regulation of these genes over SrbA-SrbB heterodimers. In contrast, *erg1*, *erg25A*, *niiA*, *alcC*, and *hem13* might be favorably regulated by SrbA-SrbB heterodimers rather than SrbA- or SrbB- homodimers. Another possibility is that the promoter of *erg1*, *erg25A*, *niiA*, *alcC*, and *hem13* might contain an SrbB-specific binding site(s) in addition to the identified SrbA SRE motif. Consequently, both SrbA and SrbB could simultaneously regulate these genes by binding at the separate promoter sites.

However, the utilization of genetic null mutants and over-expression strains in null mutant backgrounds has allowed us to pinpoint the contribution of SrbA and SrbB to expression of specific target genes (Figure 8). These target genes yield new additional insights into how *A. fumigatus* adapts and grows in the mammalian lung environment. Of particular interest is the strong reduction in Δ*srbB* growth in the murine model that was similar to the reduction in growth observed in mice inoculated with Δ*srbA*. The difference in murine survival between mice inoculated with the two strains could be due to increases in host damage caused by Δ*srbB*. For example, as SrbB is the major regulator of the ethanol fermentation alcohol dehydrogenase AlcC, one would predict an increase in inflammation in Δ*srbB* inoculated mice due to the immune suppressive effects of ethanol previously reported in our murine model [7]. Alternatively or in conjunction with increased immunopathogenesis, it may be plausible that host heme is utilized by Δ*srbB* to promote growth and host death later in infection when host damage is more severe and free heme may be available as addition of hemin to Δ*srbB* partially restored *in vitro* hypoxia growth. In addition, the further decrease in fungal burden observed in Δ*srbA*Δ*srbB* strain inoculated strongly suggests that the combination of decreased iron uptake and ergosterol biosynthesis, regulated by SrbA, and defects in carbon metabolism and heme biosynthesis, regulated by primarily by SrbB, consequently severely inhibit *in vivo* fungal growth. As all of these biological processes related to fungal metabolism/bioenergetics are impacted by oxygen availability, manipulation of *in vivo* oxygen levels may be a viable therapeutic strategy to reduce *A. fumigatus* growth *in vivo*.

In conclusion, while it is well established that fungal SREBPs are critical regulators of ergosterol biosynthesis and iron homeostasis, our analyses of SrbA and SrbB expand the known functions of these fungal virulence and antifungal drug-associated transcription factors (Figure 12). The major enrichment of genes involved in oxidoreductase activity, carbohydrate and nitrogen metabolism, and heme biosynthesis in the regulons of SrbA and SrbB presents an exciting and important area for further investigation into how these processes affect hypoxia adaptation, fungal virulence, and responses to antifungal drugs. From the big picture of the SrbA-SrbB regulons, we propose that the SrbA-SrbB genetic network allows *A. fumigatus* to “reprogram” its bioenergetics to allow invasive growth to cause disease in the mammalian lung in the face of oxygen and iron limitation. Consequently, these fungal SREBPs are much more than regulators of sterol biosynthesis, rather they are global regulators of fungal bioenergetics potential/metabolism; also an emerging theme with mammalian SREBPs [80]. Finding a mean(s) to unplug this fungal genetic network is an ongoing research goal that is expected to yield a significant therapeutic breakthrough for IPA.

## Materials and Methods

### Strains and Media

*Aspergillus fumigatus* strain CEA17 was used to construct the *ΔsrbA* mutant [36]. The wild type strain referred to in this article is strain CBS 144.89, also called CEA10. All strains are routinely grown on glucose minimal media (GMM) that contains 1% glucose, salt solution and trace minerals, at 37°C [81]. The recipe for liquid glucose minimal media is identical to that for GMM, except without agar. Liquid cultures for RNA analysis were grown under agitation (200 RPM) in baffle flasks.

### Construct Design for an *srbB* Null Mutant and *srbA* or *srbB* Over-Expression Strains

To generate an *srbB* null mutant, a 1.2 kb up- and downstream sequences were PCR-amplified from *A. fumigatus* genomic DNA (gDNA). As a selectable marker, a 3.2 kb *pyrG* from *A. parasiticus* was PCR-amplified from the plasmid pJW24. The three DNA fragments were used as a template to generate a final construct via double-joint PCR, and the PCR product was transformed to *A. fumigatus* wild type CEA17 [30]. Southern blot analysis was conducted to confirm homologous gene replacement (Figure S2). To regain *srbB* expression in the *srbB* deletion strain, a 4.1 kb DNA fragment including a *srbB* promoter and a coding sequence was PCR-amplified. As a selectable marker, a 3.0 kb *hygB* fragment was PCR-amplified from pBC-hyg plasmid DNA. These two PCR products were used as a template to generate a final reconstituted construct via a double-joint PCR [82]. The final PCR product was transformed to Δ*srbB* and transformants were screened using PCR.

A double null mutant of *srbA* and *srbB* was generated by deletion of *srbB* in the Δ*srbA* (pyrG-) strain. The *srbB* deletion construct designed to generate Δ*srbB* above was transformed to Δ*srbA*. Gene replacement in the resulting transformants were screened by PCR and verified by Southern bot analysis. To over-express *srbB* in Δ*srbA* background, either *A. fumigatus* flavohemoprotein (*flavA*(p), AFUB_099650) or *A. nidulans* glyceraldehyde-3-phosphate dehydrogenase (*gpdA*(p), AN8041) promoter was utilized. A 1 kb *flavA*(p) and a 2 kb *gpdA*(p) DNA fragment was PCR-amplified from *A. fumigatus* and *A. nidulans* gDNA. A 2.5 kb DNA fragment including the *srbB* coding region and downstream sequence was PCR-amplified from *A. fumigatus* gDNA. As a selectable marker, a 3.2 kb *pyrG* from *A. parasiticus* was PCR-amplified from the plasmid pJW24. These three PCR products were used to generate a 6.5 (*flavA*(p)) and 7.5 kb (*gpdA*(p)) final construct via double-joint PCR [82]. The final constructs were transformed into Δ*srbA* resulting in TDC43.18 (*flavA*(p)) or TDC44.2 (*gpdA*(p)) strains. Single copy integration of the *srbB*-overexpression construct in Δ*srbA* was confirmed by Southern blot analysis.

To over-express *srbA* in Δ*srbA*Δ*srbB*, *srb*A was amplified along with 1.2 kb 5′ upstream sequence from A. fumigatus wild type gDNA. Purified PCR product was transformed in the Δ*srb*AΔ*srb*B strain. Transformants were selected in hypoxia using the inability of hypoxia growth of Δ*srbA*. Over-expression of *srbA* in the resulting transformants was confirmed by qRT-PCR (Figure 8).

### Chromatin Immunoprecipitation (ChIP): Growth Conditions

For ChIP experiments, 1×10^6^ spores/mL of *Aspergillus fumigatus* strain CBS144.89 and Δ*srbA* were grown in 200 mL of liquid glucose minimal media (LGMM) in 500 mL shaking flask cultures for 24 hours. Samples were centrifuged, and 200 mg of mycelia were transferred to 100 mL pre-conditioned fresh LGMM in 250 mL Erlenmeyer flasks, and then placed in hypoxia chamber on platform shaker at 200 rpm for 4 and 12 hours hypoxia exposure. Samples were collected by vacuum filtration and transferred to cross-linking solution for ChIP experiments, or flash frozen and lyophilized for RNA extraction.

### Cross-linking

A sample of mycelia for later RNA isolation was frozen on liquid nitrogen immediately before crosslinking, and stored at −80°C. Remaining filtered samples were added to 20 mL of buffer for crosslinking (0.4 M Sucrose, 10 mM Tris-HCl, pH 8.0, 1 mM EDTA, adding 1 mM PMSF and 1% formaldehyde just before use) in a 125 mL flask for 20 min under shaking (100 rpm) at 30°C. Crosslinking was stopped by adding 1 mL of 2 M glycine, and continued shaking incubation for 10 minutes. Mycelia were collected and dried using vacuum filtration and rinsed with sterile ddH2O and transferred sample to 2 mL screw cap tube, and frozen immediately with liquid nitrogen and stored at −80°C.

### DNA Sonication

Approximately 200 mg of frozen mycelia were ground to a fine powder in a chilled mortar and pestle with liquid nitrogen added. Powder was transferred to 10 mL of ChIP lysis buffer (CLB: 50 mM HEPES pH 7.5, 150 mM NaCl, 1 mM EDTA, 1% Triton X-100, 0.1% Deoxycholate (Sigma D6750), 0.1% SDS, 1 mM PMSF, 1× fungal proteinase inhibitor cocktail (Sigma, USA)). Each sample was vortexed and then split into multiple 300 µl volume (maximum volume for sonication) 1.5 mL microfuge tubes appropriate for sonication. Samples were sonicated with Biorupter UCD-200 (Diagenode, USA) with the following condition: 30 sec ON and 30 sec OFF at power level High for a total of 30 minutes in a cold room. Ice was added every 10 minutes to ensure samples remain at 4°C. Tubes were centrifuged at 10,000 *g* for 5 minutes at 4°C. Supernatant was transferred into new tube. 30 µL was reserved as input control (IC) fraction for reverse crosslinking to verify sonication and control for ChIP and qPCR.

### Chromatin Immunoprecipitation

30 µL of Protein A Dynabeads (Dynal, Invitrogen) were used for each sample. Beads were washed twice on magnetic stand with 500 µL of CLB, with 5 minutes of slow rotation at 4°C for each. 100 µL of blocking buffer (0.1 mg BSA, 200 µg Yeast tRNA, in 1 mL of CLB) with 1 µg of antibody/100 µL (IgG (Invitrogen, Rabbit) or SrbA (Willger et al. 2008, anti-rabbit antibody) was added to the washed Dynabeads on a magnetic stand. The beads and blocking buffer were incubated 16 hours at 4°C with slow rotation to coat Dynabeads. After incubation, Dynabeads were washed twice by 500 µL of CLB as above. Immediately after removing last wash, 100 µL of sonicated sample was added to beads. Samples were incubated 16 h at 4°C with rotation. In cold room, samples were washed twice by 500 µL of CLB as above, then washed as above with 0.5 ml of LNDET (0.25 M LiCl; 1% NP40 (Nonidet P40 Substitute, USB); 1% Deoxycholate; 1 mM EDTA), and finally washed twice with 0.5 ml of Tris-EDTA (TE: 10 mM Tris-HCl, pH 8.0; 1 mM EDTA, pH 8.0). After removal of last TE wash, DNA was eluted from antibody with 50 µl of fresh elution buffer (EB: 1% SDS; 0.1 M NaHCO3; 0.2 mg/ml proteinase K; 1 mM DTT) and incubated at 65°C for 10 minutes. On a magnetic stand, supernatant was transferred to a new tube. A second elution with 50 µl of EB was performed so that the final elution volume was 100 µl. All samples were incubated for 16 hours at 65°C for reverse crosslinking.

### DNA Purification for ChIP Samples

After reverse crosslinking, samples were treated with 2.5 µg of RNase A and incubated for 30 minutes at room temperature. DNA was extracted either by using PCR purification kit (Qiagen) following the high pH protocol, or EtOH precipitation, with 50 µL as final volume. 5 µL of sample was assayed on Qubit using high sensitivity dsDNA kit (Invitrogen). To check sonication, 10 µL of IC was run on E-gel EX 2% agarose gel (Invitrogen) and fragment size ranged from 1 kb to 100 bp.

### ChIP Sequencing

ChIP-seq libraries were created following a published Illumina ChIP-seq library preparation protocol [83]. Briefly, fragmented ends were repaired, an adenine molecule was added to the repaired end, PAGE-purified adapters were added to the overhang, and the mixture amplified with primer 1.0 and primer 2.0 with an individual index tag to allow for multiplex of samples in a single lane. Samples were gel purified to obtain a size range between 200–400 bp. Libraries were validated by real time PCR, concentration was determined with Qubit (Invitrogen) and integrity was checked with an Agilent Bioanalyzer. Samples were sent to the Ohio State University sequencing facility for 76 bp paired end sequencing and four ChIP samples were indexed per lane on the Illumina HiSeq 2000. Full read data are deposited at the NCBI GEO repository under accession GSE61974.

### ChIP Sequencing Alignment, Peak Finding

Paired end reads from three independent ChIP-seq experiments (multiplexed in 1 lane each) were quality checked with fastqc (http://www.bioinformatics.babraham.ac.uk/projects/fastqc/). Reads were trimmed and cleaned of contaminating Illumina adaptors using trimmomatic [84] and aligned to the *A. fumigatus* A1163 CADRE genome from ensembl fungi, version 18 using bowtie2-2.1.0 [85]. Reads that aligned concordantly as paired ends were retained and used for peak calling. The resulting bam files were used as input for peak calling and for plotting. Peaks were called using Model-based Analysis for ChIP-Sequencing (MACS2) version 2.0.10.20131216 [45], with the subpeak calling option enabled. Peak calling was done with the wild type ChIP-seq samples and wild type input control samples using an fdr cutoff of 0.05. Results reported herein are for the combined reads from all 3 samples. Similar sets of peaks and genes were identified when each independent biological replicate was assessed separately.

### Motif Discovery and Enrichment in SrbA Regulated Genes

To find sequence motifs within ChIP-seq peaks, 100 bp centered on each of the peaks (Table S2) were selected. Multiple Em for motif elicitation (Meme) version 4.9.1 [86] was run with the minimum and maximum motif widths of 4 and 12, the zoops model and the x_branch option. The resulting 11 bp motif was reported for 54 sites. Similar motifs were found using the meme oops and anr models or using other motif discovery tools [87], [88].

### ChIP qRT-PCR

Prior to sequencing, all ChIP samples were diluted 10-fold for PCR. 1 µl of template was used in a 10 µl total volume reaction using Promega 2× GoTaq qPCR master mix and 0.4 µM of each primer. Realtime PCR was performed with 40 cycles of 95°C for 15 s and 60°C for 30 s on Mastercycler ep *realplex* PCR machine. PCR was performed in triplicate for each separate ChIP experiment using primers designed for regions identified as enriched in preliminary analysis. Three genes were chosen from this analysis as positive for enrichment in all ChIP conditions (*srbA*, *erg11A* and e*rg25A*) based on previous microarray experiments [40]. Percent input method was used to calculate the signal of enrichment of the promoter region for each gene (http://cshprotocols.cshlp.org/cgi/content/full/2009/9/pdb.prot5279 and Invitrogen website). Briefly, 100*(2^(InputCt-ChIPCt)^) was calculated for each reaction and the average and standard deviation calculated from these values. No correction for adjusted input was necessary as both templates were diluted equally prior to PCR. Lack of enrichment for at least two of the three genes, or non-amplifying PCR, was evidence for poor ChIP, and these samples were not sequenced. ChIP-qPCRs with SrbB:GFP were performed for the genes that showed significant SrbA enrichment on the promoters. ChIP samples were prepared from cultures grown under the same conditions used for ChIP-seq. All ChIP samples were diluted 10-fold for PCR. 2 µl of template was used in a 20 µl total volume reaction using SYBR green master mix (BioRad) and 0.4 µM of each primer. Fold enrichment method was used to calculate the signal of the promoter region for each gene (http://www.lifetechnologies.com/us/en/home/life-science/epigenetics-noncoding-rna-research/chromatin-remodeling/chromatin-immunoprecipitation-chip/chip-analysis.html).

### RNA Extraction for Nanostring and RNA-seq

For RNA experiments, 1×10^6^ conidia of *Aspergillus fumigatus* strain CBS144.89, Δ*srbA* and Δ*srbB* strains were grown in 50 mL of LGMM in 250 mL shaking baffle flasks for 16 hours at 300 RPM. Samples were transferred to a hypoxia chamber on platform shaker at 200 RPM for variable hypoxia exposure. Samples were collected by vacuum filtration, flash frozen in liquid nitrogen and lyophilized for RNA extraction. Lyophilized tissue was mixed with 0.2 mL of 0.5 mm glass beads (BioSpec, USA) and mixed for 30 seconds on Mini-beadbeater-16 (BioSpec, USA). Ground mycelia were suspended in 1 mL of TriSure (BioLine, USA), and incubated for 5 minutes. 200 µl of chloroform was added and mixed well, incubated two minutes. Samples were centrifuged at maximum speed for 15 minutes at 4°C. Upper layer was collected and mixed with an equal volume of 80% EtOH, and pipetted immediately to RNeasy spin column (Qiagen, USA). Columns were centrifuged one minute, flow through discarded and washed twice with kit supplied RPE buffer, and column completely dried after last wash. Filter column was transferred to RNase free 1.5 mL tube, and 200 uL of RNase free water was added an incubated for 1 minute, and then centrifuged to obtain nucleic acid. Samples were analyzed with NanoDrop ND-1000 (Thermo-Fisher).

### RNA Sample Preparation and Illumina Sequencing (RNA-Seq)

To identify transcriptionally active genes, cDNAs obtained from the fungal mycelia incubated at stated conditions were sequenced with the Illumina platform to determine transcript abundance. Samples were DNased using the RNeasy kit (Qiagen), following the protocol for DNase Digestion before RNA Cleanup. Sequencing libraries were generated using the ScriptSeq kit v2 (Epicenter) following manufacturer's directions. After each preparation step sample quality and quantity was assessed using Bioanalyzer and the Agilent RNA 6000 Nano Kit (Agilent). All cDNA libraries were sequenced (4 samples per lane) using the Illumina HiSeq2000 instrument (www.illumina.com) at the Oregon Health Sciences University Massively Parallel Sequencing Shared Resource (http://www.ohsu.edu/xd/research/research-cores/mpssr/). RNA-seq analysis was performed using the bowtie-tophat-cufflinks pipeline [85], [89], [90]. RNA-seq data is deposited at NCBI SRA under BioProject ID PRJNA240563: accession numbers SAMN02677488, SAMN02677489, SAMN02677490.

### FunCat and GO Enrichment Analysis

The FungiFun2 2.27 beta web-based server https://elbe.hki-jena.de/fungifun/fungifun.php was utilized to interrogate the functional enrichment of FunCat and gene ontologies in the respective datasets. For RNA-seq analysis, genes whose mRNA levels changed 4 fold or greater were included in the analysis. The A1163 genome selection was utilized for all analyses. Settings used for statistical significance include: significance level 0.05, significance test Hypergeometric distribution, and the Benjamini-Hochberg adjustment method.

### cDNA Synthesis and Quantitative Real-Time-PCR

*Aspergillus fumigatus* strains were cultured in liquid media (glucose-minimal-media or induction/repression minimal media) for sixteen hours, then shifted to hypoxia for the indicated times. Mycelia were harvested via vacuum filtration and lyophilized overnight prior to homogenization with 0.1 mm glass beads. Total RNA was extracted using TRisure (Bioline) according to the manufacturer's instruction and purified via RNeasy column protocol (Qiagen). Genomic DNA purification was completed with Turbo DNAse I (Ambion). A secondary genomic DNA purification was done with the Qiagen QuantiTect Reverse Transcription Kit (Qiagen), as well as oligo-DT-primed cDNA synthesis. qRT-PCR was conducted in technical duplicates except where noted. The normalized fold expression graphed in each figure represents the mean and percent error of two-to-three biological replicates as normalized to the housekeeping gene *tefA*. A no-template mRNA control was used to ensure no gDNA contamination in each analysis.

### Murine Model of IPA and In Vivo Analysis of Transcript Abundance

Virulence study for Δ*srbB*, wild type and reconstitution strains (20 animals per strain, 2 experiments) was conducted in CD1 mice (Charles Rivers, USA) using the triamcinolone infection model for *A. fumigatus* as previously described [7]. For *in* vivo RNA analysis, mouse lungs were harvested on day 2, 3 and 4 post-infection. Lungs were flash frozen in liquid nitrogen and lyophilized for 24–48 hours until lung tissue was completely dry. Tissue was processed in same manner as above for fungal RNA. Final RNA samples were combined as whole lung, after verifying fungal RNA was present in each sample by qRT-PCR.

### Fungal Burden Assay

For the determination of fungal burden, triamcinolone mouse model of IPA was utilized as described previously [7]. Briefly, mice were immunosuppressed with a single sub-cutaneous injection of steroid triamcinolone (Kenalog −10) (40 mg/kg) on day −1. Mice were inoculated with 10^6^ conidia of the respective strains in 40 µl PBS intranasally on day zero. Control mice were infected with PBS only. Lungs were collected on day +3 and were immediately frozen in liquid nitrogen. DNA was isolated from lyophilized lungs, and treated with RNAse overnight. qRT-PCR was done to determine the amount of fungal DNA in each sample by comparing with a standard curve of known concentrations as described previously [7], [61]. Data represented are the mean and standard error of 3–5 mice per group and analyzed by t-tests between 2 experimental groups.

### Ethics Statement

We carried out our animal studies in strict accordance with the recommendations in the Guide for the Care and Use of Laboratory Animals of the National Research Council (Council, 1996). The animal experimental protocol was approved by the Institutional Animal Care and Use Program (IACUC) at Montana State University Federal-Wide Assurance Number: A3637-01) and by the Institutional Animal Care and Use Committee (IACUC) at Dartmouth College (Federal-Wide Assurance Number: A3259-01).

### Nanostring Quantitation of In Vitro and In Vivo RNA

Digital counts for 60 genes (ChIP targets, housekeeping genes and other genes of interest) were adjusted for binding efficiency with background subtraction using the included positive and negative controls from the manufacturer (Nanostring Technologies, Seattle, WA, USA), as per Nanostring nCounter data analysis guidelines. Data sets were normalized to facilitate across sample comparisons using the geometric mean of 20 stably expressed genes. A subset of 12 of these genes were examined and presented herein with complete data in Table S5. Boxplots were generated in R [91].

### Growth and Biomass Production Tests

Sequentially diluted spore suspensions in 5 µl sterile water (10^2^–10^5^ conidia) were dropped on GMM plates and cultured at 37°C for 3 days in normoxia or hypoxia (1% O_2_, 5% CO_2_). In order to study biomass production, 10^8^ conidia were incubated in 200 mL liquid GMM at 37°C, 200 rpm for 2 days. Mycelia were harvested, lyophilized, and weighed. Biomass test was performed in triplicate.

### Susceptibility Test to Voriconazole (VCZ)

Susceptibility of fungal strains to VCZ was tested using either commercially available E-test strips (Biomerieux, Inc. Durham, NC) or a semi-quantitative method directly using VCZ (Sigma) solutions with appropriate concentrations. Five mL of RPMI media or GMM containing 10^5^ conidia were overlaid onto a 25 mL RPMI/GMM plate. Then, E-test strips were placed on the plate, or VCZ (Sigma) solutions were added in the center of the plate. After 2 days incubation at 37°C, clearance of fungal growth was observed and susceptibility to VCZ was decided.

### Analysis of Porphyrins

Porphyrins were extracted from powdered dried mycelia by homogenization in phosphate buffered saline and the protein content determined in an aliquot by the Bradford method. The extract was then mixed with an equal volume of acetone/concentrated HCl (97.5/2.5 v/v), centrifuged and the supernatant analyzed for porphyrins by reversed phase HPLC with fluorescence and UV detection [92] using porphyrin standards from Frontier Scientific (Logan, UT, USA)

## Supporting Information

Figure S1 **Alignment of basic HLH domains from fungal SREBPs.** Basic Helix-Loop-Helix (bHLH) domains from sterol regulatory element binding proteins (SREBPs) from *S. pombe* (Sre1 and Sre2), *C. neoformans* (Sre1), *C. albicans* (Cph2), *Aspergillus oryzae* (SclR), and *A. fumigatus* (SrbA and SrbB) were compared using Gel-Doc software. Black and grey areas represent identical and similar amino acid residues, respectively. A red arrow indicates Arg→Tyr substitution found in SREBPs differentially from other bHLH transcription factors. SpSre1/2: *S. pombe* Sre1 or Sre2, CnSre1: *C. neoformans* Sre1, CaCph2: *C. albicans* Cph2, AfSrbA/B: *A. fumigatus* SrbA or SrbB, AoSclR: *A. oryzae* SclR.
(TIF)

Figure S2 **Generation of an** ***srbB*** **null mutant.** (A). The *srbB* coding region was replaced with *Aspergillus parasiticus pyrG*. Southern blot analysis was conducted to verify the replacement of *srbB* by homologous gene recombination. The restriction enzyme, *ApaI* was used to digest genomic DNA of Δ*srbB* and wild type. A 1.1 kb DNA fragment of 5′ flank sequence of *srbB* was amplified for Southern probe. (B). Wild type and Δ*srbB* showed a 5.3 and 6.4 kb DNA fragment, respectively, which agreed with correct band sizes estimated by sequence analysis.
(TIF)

Figure S3 **Generation of the SrbB:GFP strain and localization of SrbB:GFP.** (A). SrbB tagged with GFP was expressed in *A. fumigatus* wild type. Expression of *srbB* was examined using qRT-PCR from cultures prepared as ChIP-seq samples. Compared to wild type, the SrbB:GFP strain shows similar *srbB* expression in tested conditions. (B). Localization of SrbB:GFP in germlings was observed under microscope. SrbB:GFP is localized to the nucleus (marked as arrow heads). DIC = Differential interference contrast.
(TIF)

Figure S4 **Phenotype of an** ***srbA srbB*** **double null mutant and a** ***srbA*** **-overexpression strain.** (A). Conidia of each strain were inoculated on GMM and cultured at 37°C for 2 days in normoxia or hypoxia (1% oxygen, 5% carbon dioxide). Δ*srbB* shows reduced radial growth in hypoxia compared to wild type, and Δ*srbA*Δ*srbB* does not grow in hypoxia similar to Δ*srbA*. (B). Sensitivity to voriconozole (VCZ) was tested under different concentrations 0, 0.1, and 0.2 µg/ml. Compared to wild type, Δ*srbA*Δ*srbB* shows increased sensitivity to VCZ, which phenocopies Δ*srbA*. (C). *srbA* was overexpressed in Δ*srbA*Δ*srbB* (expression was verified by qRT-PCR as shown in Figure 8), and growth of the resulting strain was studied in normoxia and hypoxia. A thousand conidia were inoculated on GMM and cultured at 37°C for 3 days. Over-expression of *srbA* restored hypoxic growth of Δ*srbA*Δ*srbB*.
(TIF)

Table S1 **Summary of ChIP-seq next generation sequencing data for each respective sample.**
(XLSX)

Table S2 **Significant peaks and associated genes with False Discovery Rate of 0.05 from SrbA ChIP-seq analyses.**
(XLSX)

Table S3 **Summary of RNA-seq FungiFun2 analyses.** Transcripts with mRNA levels increased or decreased 4 fold or greater in the *srbA* null mutant compared to wild-type are presented.
(XLSX)

Table S4 **Summary of processed RNA-seq analyses of srbA and srbB null mutants compared to wild-type at 30 and 120 minutes post-exposure to hypoxia.**
(XLSX)

Table S5 **Processed Nanostring nCounter gene expression data comparing srbA null mutant to wild-type in vitro under normoxic and hypoxic conditions and nCounter data for in vivo gene expression in a murine model of invasive pulmonary aspergillosis.**
(XLSX)

Table S6 **Summary of FungiFun2 analyses with genes changed 4 fold or greater in srbB null mutant vs. wild-type.**
(XLSX)

## References

[1] Barron MA, Madinger NE (2008) Opportunistic Fungal Infections, Part 2: *Candida* and *Aspergillus* . Infections in Medicine 25: 498–505.

[2] Brown GD, Denning DW, Gow NA, Levitz SM, Netea MG, et al (2012) Hidden killers: human fungal infections. Science translational medicine 4: 165rv113. 10.1126/scitranslmed.3004404 23253612

[3] Brown GD, Denning DW, Levitz SM (2012) Tackling human fungal infections. Science 336: 647. 22582229 10.1126/science.1222236

[4] Ben-Ami R, Lewis RE, Kontoyiannis DP (2010) Enemy of the (immunosuppressed) state: an update on the pathogenesis of *Aspergillus fumigatus* infection. British Journal of Haematology 150: 406–417. 20618330 10.1111/j.1365-2141.2010.08283.x

[5] Steinbach WJ (2013) Are We There Yet? Recent Progress in the Molecular Diagnosis and Novel Antifungal Targeting of *Aspergillus fumigatus* and Invasive Aspergillosis. PLoS Pathogens 9: e1003642. 24204250 10.1371/journal.ppat.1003642 PMC3812002

[6] Willger S, Grahl N, Cramer R (2009) *Aspergillus fumigatus* metabolism: Clues to mechanisms of in vivo fungal growth and virulence. Medical Mycology 47: S72–S79. 19253141 10.1080/13693780802455313 PMC2905159

[7] Grahl N, Puttikamonkul S, Macdonald JM, Gamcsik MP, Ngo LY, et al (2011) *In vivo* hypoxia and a fungal alcohol dehydrogenase influence the pathogenesis of invasive pulmonary aspergillosis. PLoS Pathogens 7: e1002145. 21811407 10.1371/journal.ppat.1002145 PMC3141044

[8] Tarrand JJ, Han XY, Kontoyiannis DP, May GS (2005) *Aspergillus* hyphae in infected tissue: Evidence of physiologic adaptation and effect on culture recovery. Journal of Clinical Microbiology 43: 382–386. 15634998 10.1128/JCM.43.1.382-386.2005 PMC540129

[9] Hall LA, Denning DW (1994) Oxygen Requirements of *Aspergillus* Species. Journal of Medical Microbiology 41: 311–315. 7966201 10.1099/00222615-41-5-311

[10] Brock M, Jouvion G, Droin-Bergere S, Dussurget O, Nicola MA, et al (2008) Bioluminescent *Aspergillus fumigatus*, a new tool for drug eficiency testing and *in vivo* monitoring of invasive aspergillosis. Applied and Environmental Microbiology 74: 7023–7035. 18820063 10.1128/AEM.01288-08 PMC2583481

[11] Brown JM, Wilson WR (2004) Exploiting tumour hypoxia in cancer treatment. Nature reviews Cancer 4: 437–447. 15170446 10.1038/nrc1367

[12] Koeppen M, Eckle T, Eltzschig HK (2011) The hypoxia-inflammation link and potential drug targets. Current opinion in anaesthesiology 24: 363–369. 21659868 10.1097/ACO.0b013e32834873fd PMC3259566

[13] Eltzschig HK, Carmeliet P (2011) Hypoxia and inflammation. The New England Journal of Medicine 364: 656–665. 21323543 10.1056/NEJMra0910283 PMC3930928

[14] Moeller BJ, Richardson RA, Dewhirst MW (2007) Hypoxia and radiotherapy: opportunities for improved outcomes in cancer treatment. Cancer Metastasis Reviews 26: 241–248. 17440683 10.1007/s10555-007-9056-0

[15] Grahl N, Cramer RA Jr (2010) Regulation of hypoxia adaptation: an overlooked virulence attribute of pathogenic fungi? Medical Mycology: Official Publication of the International Society for Human and Animal Mycology 48: 1–15. 10.3109/13693780902947342 PMC2898717 19462332

[16] Ernst JF, Tielker D (2009) Responses to hypoxia in fungal pathogens. Cellular Microbiology 11: 183–190. 19016786 10.1111/j.1462-5822.2008.01259.x

[17] Hsu JL, Khan MA, Sobel RA, Jiang X, Clemons KV, et al (2013) *Aspergillus fumigatus* invasion increases with progressive airway ischemia. PloS One 8: e77136. 24155924 10.1371/journal.pone.0077136 PMC3796538

[18] Chun CD, Liu OW, Madhani HD (2007) A link between virulence and homeostatic responses to hypoxia during infection by the human fungal pathogen *Cryptococcus neoformans* . PLoS Pathogens 3: e22. 17319742 10.1371/journal.ppat.0030022 PMC1803011

[19] Chang YC, Bien CM, Lee H, Espenshade PJ, Kwon-Chung KJ (2007) Sre1p, a regulator of oxygen sensing and sterol homeostasis, is required for virulence in *Cryptococcus neoformans* . Molecular microbiology 64: 614–629. 17462012 10.1111/j.1365-2958.2007.05676.x

[20] Grahl N, Shepardson KM, Chung D, Cramer RA (2012) Hypoxia and fungal pathogenesis: to air or not to air? Eukaryotic cell 11: 560–570. 22447924 10.1128/EC.00031-12 PMC3346435

[21] Bien CM, Espenshade PJ (2010) Sterol regulatory element binding proteins in fungi: hypoxic transcription factors linked to pathogenesis. Eukaryotic Cell 9: 352–359. 20118213 10.1128/EC.00358-09 PMC2837984

[22] Hua X, Wu J, Goldstein JL, Brown MS, Hobbs HH (1995) Structure of the human gene encoding sterol regulatory element binding protein-1 (SREBF1) and localization of SREBF1 and SREBF2 to chromosomes 17p11.2 and 22q13. Genomics 25: 667–673. 7759101 10.1016/0888-7543(95)80009-b

[23] Horton JD (2002) Sterol regulatory element-binding proteins: transcriptional activators of lipid synthesis. Biochemical Society transactions 30: 1091–1095. 12440980 10.1042/bst0301091

[24] Seo YK, Chong HK, Infante AM, Im SS, Xie X, et al (2009) Genome-wide analysis of SREBP-1 binding in mouse liver chromatin reveals a preference for promoter proximal binding to a new motif. Proceedings of the National Academy of Sciences of the United States of America 106: 13765–13769. 19666523 10.1073/pnas.0904246106 PMC2728968

[25] Seo YK, Jeon TI, Chong HK, Biesinger J, Xie X, et al (2011) Genome-wide localization of SREBP-2 in hepatic chromatin predicts a role in autophagy. Cell Metabolism 13: 367–375. 21459322 10.1016/j.cmet.2011.03.005 PMC3086264

[26] Reed BD, Charos AE, Szekely AM, Weissman SM, Snyder M (2008) Genome-wide occupancy of SREBP1 and its partners NFY and SP1 reveals novel functional roles and combinatorial regulation of distinct classes of genes. PLoS Genetics 4: e1000133. 18654640 10.1371/journal.pgen.1000133 PMC2478640

[27] Hughes AL, Todd BL, Espenshade PJ (2005) SREBP pathway responds to sterols and functions as an oxygen sensor in fission yeast. Cell 120: 831–842. 15797383 10.1016/j.cell.2005.01.012

[28] Todd BL, Stewart EV, Burg JS, Hughes AL, Espenshade PJ (2006) Sterol regulatory element binding protein is a principal regulator of anaerobic gene expression in fission yeast. Molecular and Cellular Biology 26: 2817–2831. 16537923 10.1128/MCB.26.7.2817-2831.2006 PMC1430309

[29] Porter JR, Burg JS, Espenshade PJ, Iglesias PA (2010) Ergosterol regulates sterol regulatory element binding protein (SREBP) cleavage in fission yeast. The Journal of Biological Chemistry 285: 41051–41061. 20959444 10.1074/jbc.M110.144337 PMC3003404

[30] Lloyd SJ, Raychaudhuri S, Espenshade PJ (2013) Subunit architecture of the Golgi Dsc E3 ligase required for sterol regulatory element-binding protein (SREBP) cleavage in fission yeast. The Journal of Biological Chemistry 288: 21043–21054. 23760507 10.1074/jbc.M113.468215 PMC3774371

[31] Cheung R, Espenshade PJ (2013) Structural requirements for sterol regulatory element-binding protein (SREBP) cleavage in fission yeast. The Journal of Biological Chemistry 288: 20351–20360. 23729666 10.1074/jbc.M113.482224 PMC3711301

[32] Porter JR, Lee CY, Espenshade PJ, Iglesias PA (2012) Regulation of SREBP during hypoxia requires Ofd1-mediated control of both DNA binding and degradation. Molecular Biology of the Cell 23: 3764–3774. 22833559 10.1091/mbc.E12-06-0451 PMC3442422

[33] Stewart EV, Lloyd SJ, Burg JS, Nwosu CC, Lintner RE, et al (2012) Yeast sterol regulatory element-binding protein (SREBP) cleavage requires Cdc48 and Dsc5, a ubiquitin regulatory X domain-containing subunit of the Golgi Dsc E3 ligase. The Journal of Biological Chemistry 287: 672–681. 22086920 10.1074/jbc.M111.317370 PMC3249121

[34] Hughes BT, Nwosu CC, Espenshade PJ (2009) Degradation of sterol regulatory element-binding protein precursor requires the endoplasmic reticulum-associated degradation components Ubc7 and Hrd1 in fission yeast. The Journal of Biological Chemistry 284: 20512–20521. 19520858 10.1074/jbc.M109.002436 PMC2742815

[35] Hughes BT, Espenshade PJ (2008) Oxygen-regulated degradation of fission yeast SREBP by Ofd1, a prolyl hydroxylase family member. The EMBO Journal 27: 1491–1501. 18418381 10.1038/emboj.2008.83 PMC2396400

[36] Willger SD, Puttikamonkul S, Kim KH, Burritt JB, Grahl N, et al (2008) A sterol-regulatory element binding protein is required for cell polarity, hypoxia adaptation, azole drug resistance, and virulence in *Aspergillus fumigatus* . PLoS Pathogens 4: e1000200. 18989462 10.1371/journal.ppat.1000200 PMC2572145

[37] Willger SD, Cornish EJ, Chung D, Fleming BA, Lehmann MM, et al (2012) Dsc orthologs are required for hypoxia adaptation, triazole drug responses, and fungal virulence in Aspergillus fumigatus. Eukaryotic Cell 11: 1557–1567. 23104569 10.1128/EC.00252-12 PMC3536281

[38] Blatzer M, Barker BM, Willger SD, Beckmann N, Blosser SJ, et al (2011) SREBP coordinates iron and ergosterol homeostasis to mediate triazole drug and hypoxia responses in the human fungal pathogen *Aspergillus fumigatus* . PLoS Genetics 7: e1002374. 22144905 10.1371/journal.pgen.1002374 PMC3228822

[39] Chang YC, Ingavale SS, Bien C, Espenshade P, Kwon-Chung KJ (2009) Conservation of the sterol regulatory element-binding protein pathway and its pathobiological importance in *Cryptococcus neoformans* . Eukaryotic Cell 8: 1770–1779. 19749173 10.1128/EC.00207-09 PMC2772393

[40] Barker BM, Kroll K, Vodisch M, Mazurie A, Kniemeyer O, et al (2012) Transcriptomic and proteomic analyses of the *Aspergillus fumigatus* hypoxia response using an oxygen-controlled fermenter. BMC genomics 13: 62. 22309491 10.1186/1471-2164-13-62 PMC3293747

[41] Butler G (2013) Hypoxia and gene expression in eukaryotic microbes. Annual Review of Microbiology 67: 291–312. 10.1146/annurev-micro-092412-155658 23808338

[42] Sailsbery JK, Atchley WR, Dean RA (2012) Phylogenetic analysis and classification of the fungal bHLH domain. Molecular Biology and Evolution 29: 1301–1318. 22114358 10.1093/molbev/msr288 PMC3339315

[43] Davies BS, Rine J (2006) A role for sterol levels in oxygen sensing in *Saccharomyces cerevisiae* . Genetics 174: 191–201. 16783004 10.1534/genetics.106.059964 PMC1569814

[44] Losada L, Barker BM, Pakala S, Joardar V, Zafar N, et al (2014) Large-scale transcriptional response to hypoxia in *Aspergillus fumigatus* observed using RNAseq identifies a novel hypoxia regulated ncRNA. Mycopathologia [Epub ahead of print]. 10.1007/s11046-014-9779-8 PMC4239182 24996522

[45] Zhang Y, Liu T, Meyer CA, Eeckhoute J, Johnson DS, et al (2008) Model-based analysis of ChIP-Seq (MACS). Genome Biology 9: R137. 18798982 10.1186/gb-2008-9-9-r137 PMC2592715

[46] Grant CE, Bailey TL, Noble WS (2011) FIMO: scanning for occurrences of a given motif. Bioinformatics 27: 1017–1018. 21330290 10.1093/bioinformatics/btr064 PMC3065696

[47] Linde J, Hortschansky P, Fazius E, Brakhage AA, Guthke R, et al (2012) Regulatory interactions for iron homeostasis in *Aspergillus fumigatus* inferred by a Systems Biology approach. BMC Systems Biology 6: 6. 22260221 10.1186/1752-0509-6-6 PMC3305660

[48] Yokoyama C, Wang X, Briggs MR, Admon A, Wu J, et al (1993) SREBP-1, a basic-helix-loop-helix-leucine zipper protein that controls transcription of the low density lipoprotein receptor gene. Cell 75: 187–197. 8402897

[49] Kim JB, Spotts GD, Halvorsen YD, Shih HM, Ellenberger T, et al (1995) Dual DNA binding specificity of ADD1/SREBP1 controlled by a single amino acid in the basic helix-loop-helix domain. Molecular and Cellular Biology 15: 2582–2588. 7739539 10.1128/mcb.15.5.2582 PMC230488

[50] Perez JC, Kumamoto CA, Johnson AD (2013) *Candida albicans* commensalism and pathogenicity are intertwined traits directed by a tightly knit transcriptional regulatory circuit. PLoS Biology 11: e1001510. 23526879 10.1371/journal.pbio.1001510 PMC3601966

[51] Priebe S, Linde J, Albrecht D, Guthke R, Brakhage AA (2011) FungiFun: a web-based application for functional categorization of fungal genes and proteins. Fungal Genetics and Biology 48: 353–358. 21073976 10.1016/j.fgb.2010.11.001

[52] Geiss GK, Bumgarner RE, Birditt B, Dahl T, Dowidar N, et al (2008) Direct multiplexed measurement of gene expression with color-coded probe pairs. Nature Biotechnology 26: 317–325. 10.1038/nbt1385 18278033

[53] Malkov VA, Serikawa KA, Balantac N, Watters J, Geiss G, et al (2009) Multiplexed measurements of gene signatures in different analytes using the Nanostring nCounter Assay System. BMC Research Notes 2: 80. 19426535 10.1186/1756-0500-2-80 PMC2688518

[54] Schrettl M, Kim HS, Eisendle M, Kragl C, Nierman WC, et al (2008) SreA-mediated iron regulation in *Aspergillus fumigatus* . Molecular Microbiology 70: 27–43. 18721228 10.1111/j.1365-2958.2008.06376.x PMC2610380

[55] Znaidi S, Nesseir A, Chauvel M, Rossignol T, d'Enfert C (2013) A comprehensive functional portrait of two heat shock factor-type transcriptional regulators involved in *Candida albicans* morphogenesis and virulence. PLoS Pathogens 9: e1003519. 23966855 10.1371/journal.ppat.1003519 PMC3744398

[56] Zagorec M, Buhler JM, Treich I, Keng T, Guarente L, et al (1988) Isolation, sequence, and regulation by oxygen of the yeast HEM13 gene coding for coproporphyrinogen oxidase. The Journal of Biological Chemistry 263: 9718–9724. 2838478

[57] Keng T (1992) HAP1 and ROX1 form a regulatory pathway in the repression of HEM13 transcription in *Saccharomyces cerevisiae* . Molecular and Cellular Biology 12: 2616–2623. 1588959 10.1128/mcb.12.6.2616-2623.1992 PMC364455

[58] Soriani FM, Malavazi I, Ferreira MED, Savoldi M, Kress MRV, et al (2008) Functional characterization of the *Aspergillus fumigatus* CRZ1 homologue, CrzA. Molecular Microbiology 67: 1274–1291. 18298443 10.1111/j.1365-2958.2008.06122.x

[59] Blosser SJ, Merriman B, Grahl N, Chung D, Cramer RA (2014) Two C4-sterol methyl oxidases (Erg25) catalyze ergosterol intermediate demethylation and impact environmental stress adaptation in *Aspergillus fumigatus* . Microbiology DOI: 10.1099/mic.0.080440-0 10.1099/mic.0.080440-0 PMC4219106 25107308

[60] Zhou S, Fushinobu S, Kim SW, Nakanishi Y, Maruyama J, et al (2011) Functional analysis and subcellular location of two flavohemoglobins from *Aspergillus oryzae* . Fungal Genetics and Biology 48: 200–207. 20817113 10.1016/j.fgb.2010.08.011

[61] Li H, Barker BM, Grahl N, Puttikamonkul S, Bell JD, et al (2011) The small GTPase RacA mediates intracellular reactive oxygen species production, polarized growth, and virulence in the human fungal pathogen *Aspergillus fumigatus* . Eukaryotic Cell 10: 174–186. 21183690 10.1128/EC.00288-10 PMC3067399

[62] Borneman AR, Gianoulis TA, Zhang ZD, Yu H, Rozowsky J, et al (2007) Divergence of transcription factor binding sites across related yeast species. Science 317: 815–819. 17690298 10.1126/science.1140748

[63] Odom DT, Dowell RD, Jacobsen ES, Gordon W, Danford TW, et al (2007) Tissue-specific transcriptional regulation has diverged significantly between human and mouse. Nature Genetics 39: 730–732. 17529977 10.1038/ng2047 PMC3797512

[64] Schmidt D, Wilson MD, Ballester B, Schwalie PC, Brown GD, et al (2010) Five-vertebrate ChIP-seq reveals the evolutionary dynamics of transcription factor binding. Science 328: 1036–1040. 20378774 10.1126/science.1186176 PMC3008766

[65] Tuch BB, Galgoczy DJ, Hernday AD, Li H, Johnson AD (2008) The evolution of combinatorial gene regulation in fungi. PLoS Biology 6: e38. 18303948 10.1371/journal.pbio.0060038 PMC2253631

[66] Hughes TR, de Boer CG (2013) Mapping yeast transcriptional networks. Genetics 195: 9–36. 24018767 10.1534/genetics.113.153262 PMC3761317

[67] Zitomer RS, Lowry CV (1992) Regulation of gene expression by oxygen in *Saccharomyces cerevisiae* . Microbiological Reviews 56: 1–11. 1579104 10.1128/mr.56.1.1-11.1992 PMC372851

[68] Hon T, Dodd A, Dirmeier R, Gorman N, Sinclair PR, et al (2003) A mechanism of oxygen sensing in yeast. Multiple oxygen-responsive steps in the heme biosynthetic pathway affect Hap1 activity. The Journal of Biological Chemistry 278: 50771–50780. 14512429 10.1074/jbc.M303677200

[69] Hickman MJ, Winston F (2007) Heme levels switch the function of Hap1 of *Saccharomyces cerevisiae* between transcriptional activator and transcriptional repressor. Molecular and Cellular Biology 27: 7414–7424. 17785431 10.1128/MCB.00887-07 PMC2169065

[70] Thorsness M, Schafer W, D'Ari L, Rine J (1989) Positive and negative transcriptional control by heme of genes encoding 3-hydroxy-3-methylglutaryl coenzyme A reductase in *Saccharomyces cerevisiae* . Molecular and Cellular Biology 9: 5702–5712. 2685574 10.1128/mcb.9.12.5702-5712.1989 PMC363742

[71] Jin FJ, Takahashi T, Matsushima K, Hara S, Shinohara Y, et al (2011) SclR, a basic helix-loop-helix transcription factor, regulates hyphal morphology and promotes sclerotial formation in *Aspergillus oryzae* . Eukaryotic cell 10: 945–955. 21551246 10.1128/EC.00013-11 PMC3147411

[72] Rosenbach A, Dignard D, Pierce JV, Whiteway M, Kumamoto CA (2010) Adaptations of *Candida albicans* for growth in the mammalian intestinal tract. Eukaryotic Cell 9: 1075–1086. 20435697 10.1128/EC.00034-10 PMC2901676

[73] Nobile CJ, Fox EP, Nett JE, Sorrells TR, Mitrovich QM, et al (2012) A recently evolved transcriptional network controls biofilm development in *Candida albicans* . Cell 148: 126–138. 22265407 10.1016/j.cell.2011.10.048 PMC3266547

[74] Nett JE, Lepak AJ, Marchillo K, Andes DR (2009) Time course global gene expression analysis of an *in vivo Candida* biofilm. The Journal of Infectious Diseases 200: 307–313. 19527170 10.1086/599838 PMC3159582

[75] Lane S, Zhou S, Pan T, Dai Q, Liu H (2001) The basic helix-loop-helix transcription factor Cph2 regulates hyphal development in *Candida albicans* partly via TEC1. Molecular and Biology 21: 6418–6428. 10.1128/MCB.21.19.6418-6428.2001 PMC99789 11533231

[76] Sellam A, van het Hoog M, Tebbji F, Beaurepaire C, Whiteway M, et al (2014) Modeling the transcriptional regulatory network that controls the early hypoxic response in *Candida albicans* . Eukaryot Cell 13: 675–690. 24681685 10.1128/EC.00292-13 PMC4060469

[77] Datta S, Osborne TF (2005) Activation domains from both monomers contribute to transcriptional stimulation by sterol regulatory element-binding protein dimers. The Journal of Biological Chemistry 280: 3338–3345. 15550381 10.1074/jbc.M411222200

[78] Zoumi A, Datta S, Liaw LH, Wu CJ, Manthripragada G, et al (2005) Spatial distribution and function of sterol regulatory element-binding protein 1a and 2 homo- and heterodimers by in vivo two-photon imaging and spectroscopy fluorescence resonance energy transfer. Molecular and Cellular Biology 25: 2946–2956. 15798184 10.1128/MCB.25.8.2946-2956.2005 PMC1069603

[79] Robinson KA, Lopes JM (2000) SURVEY AND SUMMARY: *Saccharomyces cerevisiae* basic helix-loop-helix proteins regulate diverse biological processes. Nucleic Acids Research 28: 1499–1505. 10710415 10.1093/nar/28.7.1499 PMC102793

[80] Shao W, Espenshade PJ (2012) Expanding roles for SREBP in metabolism. Cell Metabolism 16: 414–419. 23000402 10.1016/j.cmet.2012.09.002 PMC3466394

[81] Shimizu K, Keller NP (2001) Genetic involvement of a cAMP-dependent protein kinase in a g protein signaling pathway regulating morphological and chemical transitions in *Aspergillus nidulans* . Genetics 157: 591–600. 11156981 10.1093/genetics/157.2.591 PMC1461531

[82] Yu JH, Hamari Z, Han KH, Seo JA, Reyes-Dominguez Y, et al (2004) Double-joint PCR: a PCR-based molecular tool for gene manipulations in filamentous fungi. Fungal genetics and biology 41: 973–981. 15465386 10.1016/j.fgb.2004.08.001

[83] Quail MA, Kozarewa I, Smith F, Scally A, Stephens PJ, et al (2008) A large genome center's improvements to the Illumina sequencing system. Nature Methods 5: 1005–1010. 19034268 10.1038/nmeth.1270 PMC2610436

[84] Lohse M, Bolger AM, Nagel A, Fernie AR, Lunn JE, et al (2012) RobiNA: a user-friendly, integrated software solution for RNA-Seq-based transcriptomics. Nucleic Acids Research 40: W622–627. 22684630 10.1093/nar/gks540 PMC3394330

[85] Langmead B, Trapnell C, Pop M, Salzberg SL (2009) Ultrafast and memory-efficient alignment of short DNA sequences to the human genome. Genome Biology 10: R25. 19261174 10.1186/gb-2009-10-3-r25 PMC2690996

[86] Bailey TL, Elkan C (1994) Fitting a mixture model by expectation maximization to discover motifs in biopolymers. Proceedings/International Conference on Intelligent Systems for Molecular Biology; ISMB International Conference on Intelligent Systems for Molecular Biology 2: 28–36. 7584402

[87] Carlson JM, Chakravarty A, DeZiel CE, Gross RH (2007) SCOPE: a web server for practical de novo motif discovery. Nucleic Acids Research 35: W259–264. 17485471 10.1093/nar/gkm310 PMC1933170

[88] Pavesi G, Mereghetti P, Mauri G, Pesole G (2004) Weeder Web: discovery of transcription factor binding sites in a set of sequences from co-regulated genes. Nucleic Acids Research 32: W199–203. 15215380 10.1093/nar/gkh465 PMC441603

[89] Roberts A, Trapnell C, Donaghey J, Rinn JL, Pachter L (2011) Improving RNA-Seq expression estimates by correcting for fragment bias. Genome Biology 12: R22. 21410973 10.1186/gb-2011-12-3-r22 PMC3129672

[90] Kim D, Pertea G, Trapnell C, Pimentel H, Kelley R, et al (2013) TopHat2: accurate alignment of transcriptomes in the presence of insertions, deletions and gene fusions. Genome Biology 14: R36. 23618408 10.1186/gb-2013-14-4-r36 PMC4053844

[91] RDevelopmentCoreTeam (2011) R: A language and environment for statistical computing. Vienna: R Foundation for Statistical Computing.

[92] Bonkovsky HL, Wood SG, Howell SK, Sinclair PR, Lincoln B, et al (1986) High-performance liquid chromatographic separation and quantitation of tetrapyrroles from biological materials. Anal Biochem 155: 56–64. 3717559 10.1016/0003-2697(86)90224-1

