# Correction: ChIP-seq and In Vivo Transcriptome Analyses of the *Aspergillus fumigatus* SREBP SrbA Reveals a New Regulator of the Fungal Hypoxia Response and Virulence

**DOI:** 10.1371/journal.ppat.1004576

**Published:** 2014-11-24

**Authors:** The PLOS Pathogens Staff

## Notice of Republication

This article was republished on November 10, 2014, to correct errors in the online version only. The two affiliations for the second author, Bridget M. Barker, were omitted. These are now correct in the online version and are the following:

Department of Microbiology and Immunology, Montana State University, Bozeman, Montana, United States of America

Current address: Pathogen Genomics Division, Translational Genomics Institute and Northern Arizona University, Flagstaff, Arizona, United States of America

There was no correction necessary to the PDF version.
