# Supplementary figures and images for: ChIP-seq and In Vivo Transcriptome Analyses of the Aspergillus fumigatus SREBP SrbA Reveals a New Regulator of the Fungal Hypoxia Response and Virulence
Source: PLoS Pathog. 2014 Nov 6;10(11):e1004487. doi: 10.1371/journal.ppat.1004487 (PMC4223079; doi:10.1371/journal.ppat.1004487)

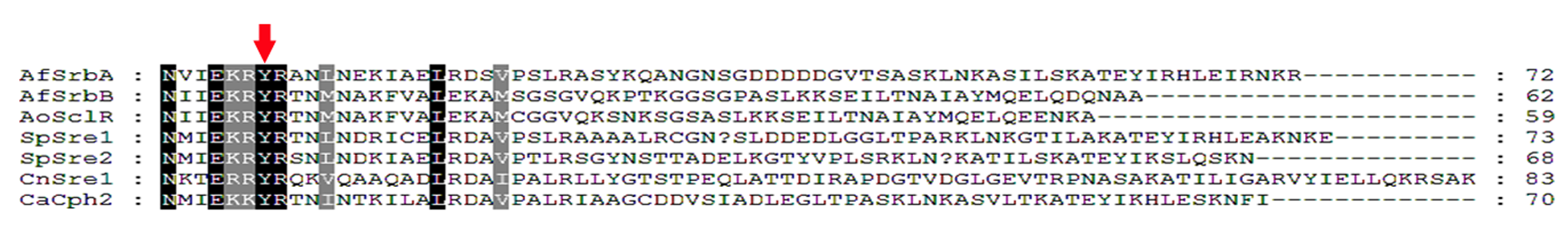

Supplement: Figure S1 — Alignment of basic HLH domains from fungal SREBPs. Basic Helix-Loop-Helix (bHLH) domains from sterol regulatory element binding proteins (SREBPs) from S. pombe (Sre1 and Sre2), C. neoformans (Sre1), C. albicans (Cph2), Aspergillus oryzae (SclR), and A. fumigatus (SrbA and SrbB) were compared using Gel-Doc software. Black and grey areas represent identical and similar amino acid residues, respectively. A red arrow indicates Arg→Tyr substitution found in SREBPs differentially from other bHLH transcription factors. SpSre1/2: S. pombe Sre1 or Sre2, CnSre1: C. neoformans Sre1, CaCph2: C. albicans Cph2, AfSrbA/B: A. fumigatus SrbA or SrbB, AoSclR: A. oryzae SclR. (TIF) [file ppat.1004487.s001.tif]

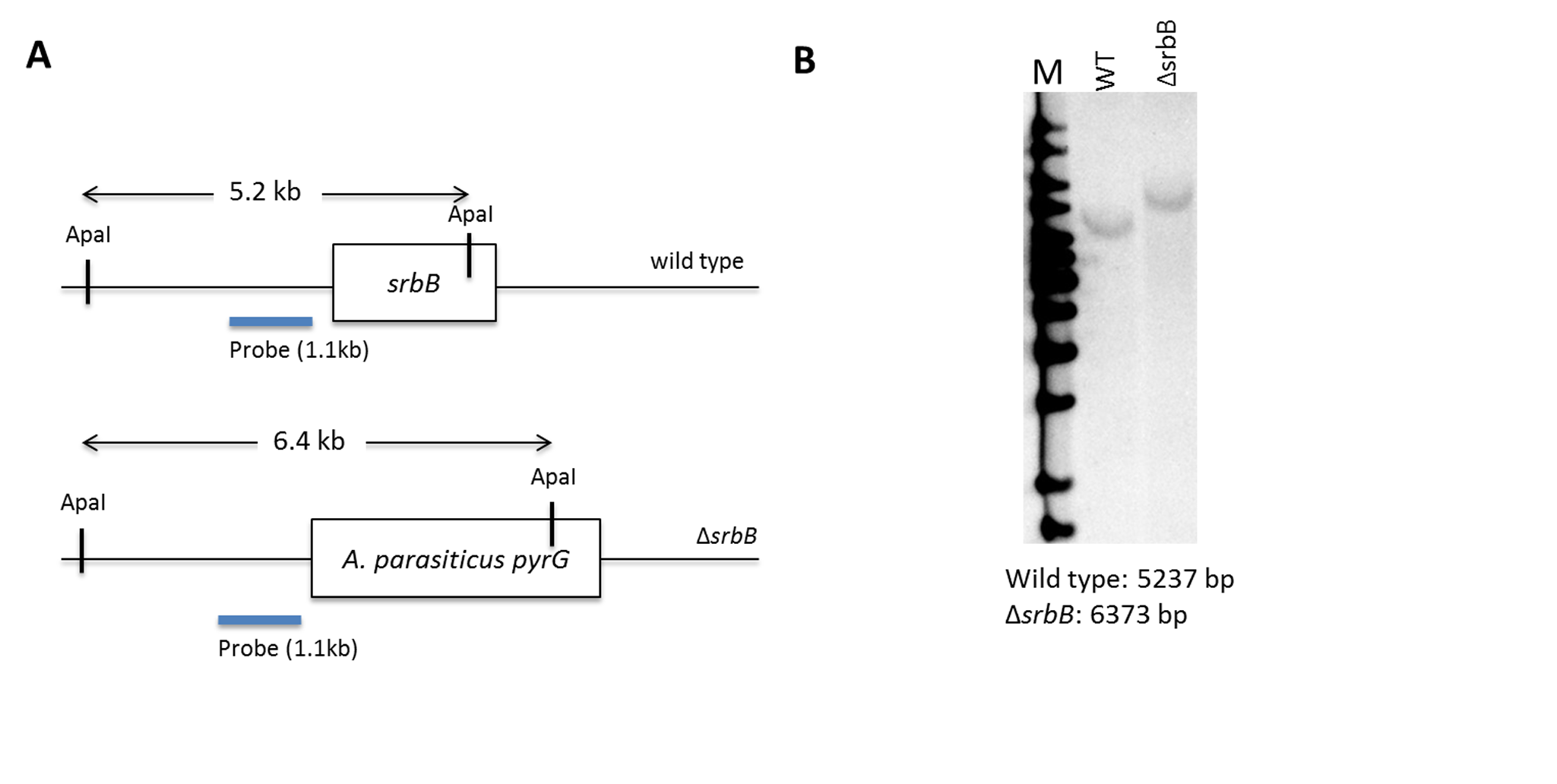

Supplement: Figure S2 — Generation of an srbB null mutant. (A). The srbB coding region was replaced with Aspergillus parasiticus pyrG. Southern blot analysis was conducted to verify the replacement of srbB by homologous gene recombination. The restriction enzyme, ApaI was used to digest genomic DNA of ΔsrbB and wild type. A 1.1 kb DNA fragment of 5′ flank sequence of srbB was amplified for Southern probe. (B). Wild type and ΔsrbB showed a 5.3 and 6.4 kb DNA fragment, respectively, which agreed with correct band sizes estimated by sequence analysis. (TIF) [file ppat.1004487.s002.tif]

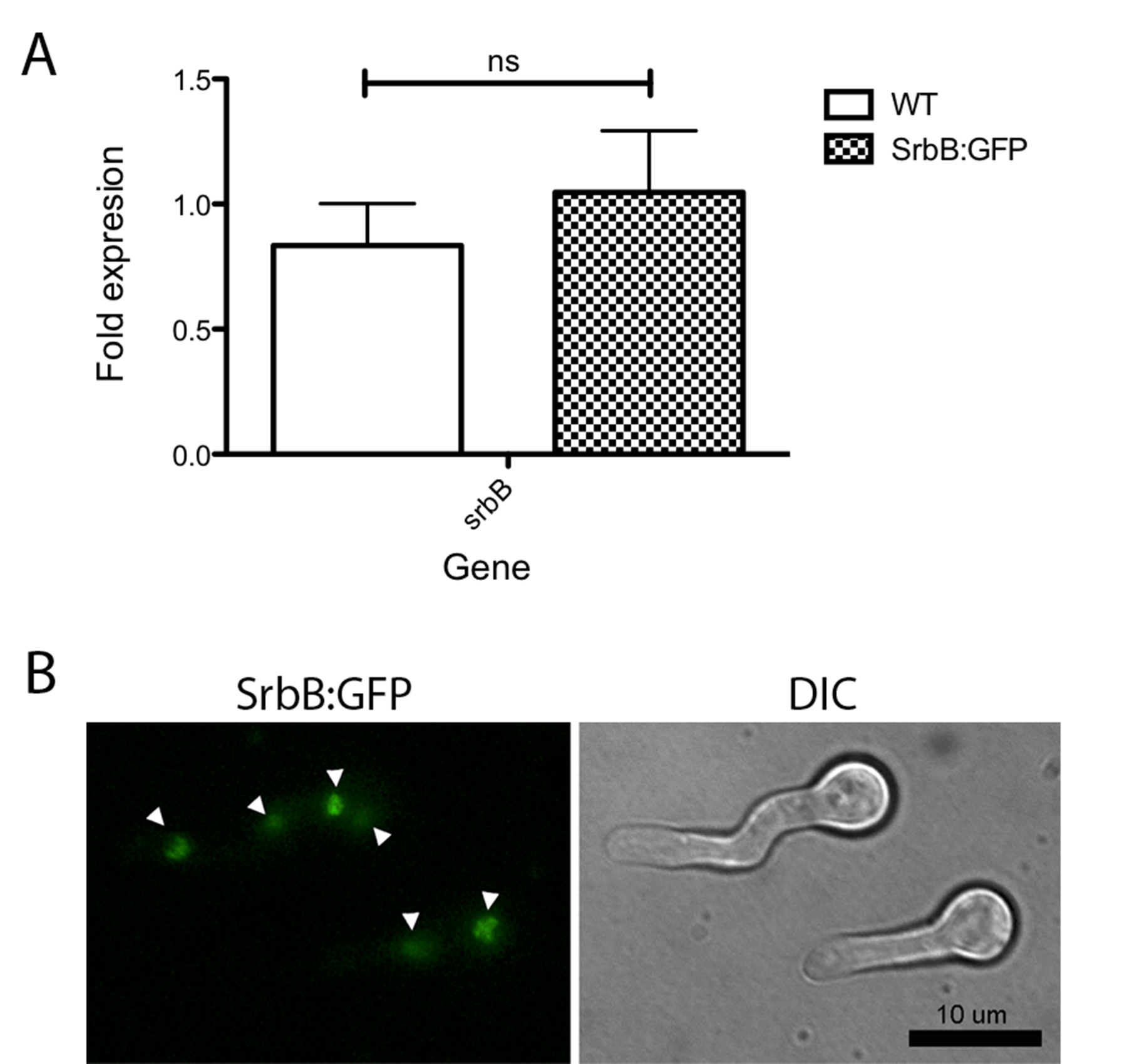

Supplement: Figure S3 — Generation of the SrbB:GFP strain and localization of SrbB:GFP. (A). SrbB tagged with GFP was expressed in A. fumigatus wild type. Expression of srbB was examined using qRT-PCR from cultures prepared as ChIP-seq samples. Compared to wild type, the SrbB:GFP strain shows similar srbB expression in tested conditions. (B). Localization of SrbB:GFP in germlings was observed under microscope. SrbB:GFP is localized to the nucleus (marked as arrow heads). DIC = Differential interference contrast. (TIF) [file ppat.1004487.s003.tif]

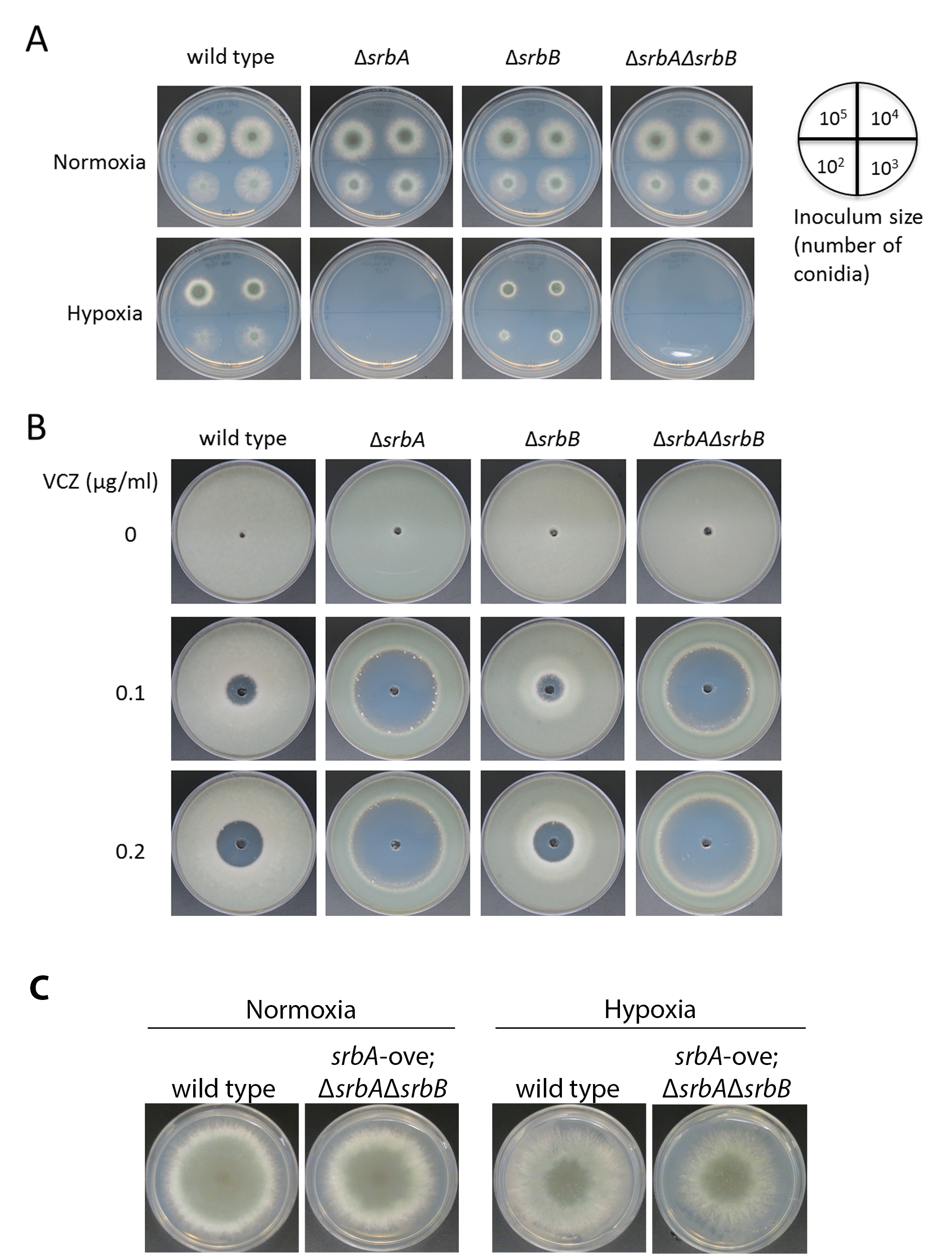

Supplement: Figure S4 — Phenotype of an srbA srbB double null mutant and a srbA -overexpression strain. (A). Conidia of each strain were inoculated on GMM and cultured at 37°C for 2 days in normoxia or hypoxia (1% oxygen, 5% carbon dioxide). ΔsrbB shows reduced radial growth in hypoxia compared to wild type, and ΔsrbAΔsrbB does not grow in hypoxia similar to ΔsrbA. (B). Sensitivity to voriconozole (VCZ) was tested under different concentrations 0, 0.1, and 0.2 µg/ml. Compared to wild type, ΔsrbAΔsrbB shows increased sensitivity to VCZ, which phenocopies ΔsrbA. (C). srbA was overexpressed in ΔsrbAΔsrbB (expression was verified by qRT-PCR as shown in Figure 8), and growth of the resulting strain was studied in normoxia and hypoxia. A thousand conidia were inoculated on GMM and cultured at 37°C for 3 days. Over-expression of srbA restored hypoxic growth of ΔsrbAΔsrbB. (TIF) [file ppat.1004487.s004.tif]
